# Validating the Cyc2 Neutrophilic Iron Oxidation Pathway Using Meta-omics of *Zetaproteobacteria* Iron Mats at Marine Hydrothermal Vents

**DOI:** 10.1128/mSystems.00553-19

**Published:** 2020-02-18

**Authors:** Sean M. McAllister, Shawn W. Polson, David A. Butterfield, Brian T. Glazer, Jason B. Sylvan, Clara S. Chan

**Affiliations:** a School of Marine Science and Policy, University of Delaware, Newark, Delaware, USA; b Center for Bioinformatics and Computational Biology, University of Delaware, Newark, Delaware, USA; c Joint Institute for the Study of Atmosphere and Ocean, University of Washington, Seattle, Washington, USA; d Pacific Marine Environmental Laboratory, National Oceanic and Atmospheric Administration, Seattle, Washington, USA; e Department of Oceanography, University of Hawai’i, Honolulu, Hawai’i, USA; f Department of Oceanography, Texas A&M University, College Station, Texas, USA; g Department of Earth Sciences, University of Delaware, Newark, Delaware, USA; University of Tennessee at Knoxville

**Keywords:** Cyc2 Fe oxidation pathway, biogeochemistry, environmental microbiology, hydrothermal vent, iron cycling, iron oxidizers, metagenomics, metatranscriptomics, microbial ecology, *Zetaproteobacteria*

## Abstract

*Zetaproteobacteria* create extensive iron (Fe) oxide mats at marine hydrothermal vents, making them an ideal model for microbial Fe oxidation at circumneutral pH. Comparison of neutrophilic Fe oxidizer isolate genomes has revealed a hypothetical Fe oxidation pathway, featuring a homolog of the Fe oxidase Cyc2 from Acidithiobacillus ferrooxidans. However, Cyc2 function is not well verified in neutrophilic Fe oxidizers, particularly in Fe-oxidizing environments. Toward this, we analyzed genomes and metatranscriptomes of *Zetaproteobacteria*, using 53 new high-quality metagenome-assembled genomes reconstructed from Fe mats at Mid-Atlantic Ridge, Mariana Backarc, and Loihi Seamount (Hawaii) hydrothermal vents. Phylogenetic analysis demonstrated conservation of Cyc2 sequences among most neutrophilic Fe oxidizers, suggesting a common function. We confirmed the widespread distribution of *cyc2* and other model Fe oxidation pathway genes across all represented *Zetaproteobacteria* lineages. High expression of these genes was observed in diverse *Zetaproteobacteria* under multiple environmental conditions and in incubations. The putative Fe oxidase gene *cyc2* was highly expressed *in situ*, often as the top expressed gene. The *cyc2* gene showed increased expression in Fe(II)-amended incubations, with corresponding increases in carbon fixation and central metabolism gene expression. These results substantiate the Cyc2-based Fe oxidation pathway in neutrophiles and demonstrate its significance in marine Fe-mineralizing environments.

**IMPORTANCE** Iron oxides are important components of our soil, water supplies, and ecosystems, as they sequester nutrients, carbon, and metals. Microorganisms can form iron oxides, but it is unclear whether this is a significant mechanism in the environment. Unlike other major microbial energy metabolisms, there is no marker gene for iron oxidation, hindering our ability to track these microbes. Here, we investigate a promising possible iron oxidation gene, *cyc2*, in iron-rich hydrothermal vents, where iron-oxidizing microbes dominate. We pieced together diverse *Zetaproteobacteria* genomes, compared these genomes, and analyzed expression of *cyc2* and other hypothetical iron oxidation genes. We show that *cyc2* is widespread among iron oxidizers and is highly expressed and potentially regulated, making it a good marker for the capacity for iron oxidation and potentially a marker for activity. These findings will help us understand and potentially quantify the impacts of neutrophilic iron oxidizers in a wide variety of marine and terrestrial environments.

## Abstract (precis)

Iron oxides are important components of our soil, water supplies, and ecosystems, as they sequester nutrients, carbon, and metals. Microorganisms can form iron oxides, but it is unclear whether this is a significant mechanism in the environment. Unlike other major microbial energy metabolisms, there is no marker gene for iron oxidation, hindering our ability to track these microbes. Here, we investigate a promising possible iron oxidation gene, *cyc2*, in iron-rich hydrothermal vents, where iron-oxidizing microbes dominate. We pieced together diverse *Zetaproteobacteria* genomes, compared these genomes, and analyzed expression of *cyc2* and other hypothetical iron oxidation genes. We show that *cyc2* is widespread among iron oxidizers and is highly expressed and potentially regulated, making it a good marker for the capacity for iron oxidation and potentially a marker for activity. These findings will help us understand and potentially quantify the impacts of neutrophilic iron oxidizers in a wide variety of marine and terrestrial environments.

## INTRODUCTION

Neutrophilic Fe-oxidizing microbes are common in marine and terrestrial environments (1), precipitating reactive Fe oxyhydroxides that sequester organic carbon, phosphate, arsenic, and many other metals (2–4). However, it has been difficult to study the effects of neutrophilic Fe oxidation in natural systems due to myriad challenges that have slowed the discovery of genetic markers of neutrophilic Fe oxidation. These Fe oxidizers are difficult to culture, so only recently have we obtained enough isolate genomes to deduce hypothetical neutrophilic Fe-oxidizing pathways. Comparative genomics has led to multiple proposed pathways, each involving an outer membrane cytochrome (5–7). However, only one pathway is present in all well-established neutrophilic Fe-oxidizing isolates (*Zetaproteobacteria* and *Gallionellaceae*), centering on a fused cytochrome-porin, Cyc2 (7–10). Yet, beyond comparative genomics, we lack evidence of the Cyc2 pathway function in neutrophilic Fe oxidizers, particularly the uncultured Fe oxidizers that dominate natural Fe systems.

The Cyc2 pathway in the neutrophilic Fe oxidizers is modeled after the Fe oxidation pathways found in acidophilic Fe oxidizers Acidithiobacillus ferrooxidans and *Leptospirillum* sp., where Cyc2 Fe oxidase function has been verified (11, 12). Weak homologs to *cyc2* from these organisms were first found in the genomes of the *Gallionellaceae* Sideroxydans lithotrophicus ES-1 and Gallionella capsiferriformans ES-2 (13). The genome of *Zetaproteobacteria* type strain Mariprofundus ferrooxydans PV-1, on the other hand, lacked homologs to known Fe oxidation genes until a proteome study discovered that *cyc2* was in fact carried by PV-1 but missing from the draft genome (14). Subsequently, *cyc2* homologs were found within the few *Zetaproteobacteria* lineages with genomic representation (9, 15). Despite the identification of *cyc2*-like genes, low amino acid sequence homology (20% sequence identity between PV-1 and *A. ferrooxidans* Cyc2) suggests that their function is speculative and needs to be validated. Without a means of testing this function biochemically or genetically, we focus on more comprehensive comparative genomics and expression in Fe-oxidizing environments.

To this end, we turned to *Zetaproteobacteria* in natural Fe microbial mats. The *Zetaproteobacteria* discovered to date are all considered to be Fe oxidizers, since every isolate grows by Fe oxidation and uncultured *Zetaproteobacteria* are typically found in Fe-oxidizing environments (10, 16–21). The *Zetaproteobacteria* are often the dominant organisms in marine hydrothermal Fe mats (22–25), where they play a key role in mat formation (26). This abundance and ubiquity in Fe-oxidizing mats make *Zetaproteobacteria* ideal for study through metagenomic and metatranscriptomic approaches. Furthermore, their taxonomic diversity allows for a robust comparative genomics study. We sampled paired metagenomes and metatranscriptomes from three hydrothermal venting regions: Loihi Seamount, the Mid-Atlantic Ridge (MAR) (Rainbow, TAG, and Snake Pit vents), and the Mariana Backarc (Urashima vent field). Recovery of high-quality metagenome-assembled genomes (MAGs) allowed us to improve the limited genomic representation of the *Zetaproteobacteria* (see reference 10 for a summary of genomic representation prior to this study). Using the MAGs for reference mapping, we explored *in situ* environmental expression of the *Zetaproteobacteria* within undisturbed natural Fe mats. In addition, we examined expression responses to Fe(II) using shipboard Fe(II) amendment experiments. With these results, we assess and update the model neutrophilic Fe oxidation pathway expressed in natural environments.

This article was submitted to an online preprint archive (27).

## RESULTS

### Microbial Fe mat sampling and geochemistry.

Over three expeditions, we sampled a wide diversity of Fe microbial mats (see Table S1 in the supplemental material). Sampled mats varied in their physical setting, with meter-scale Loihi mats found in direct and indirect flow from vent fissures, mat mounds on the scale of tens of centimeters at the MAR at the diffuse-venting periphery of black smoker fields, and the mats at the Mariana Backarc Urashima vent fields covering a 7-m-tall Fe chimney (Fig. S1 and Text S1). Temperatures ranged from 10 to 63°C, while geochemical conditions also varied widely, notably concentrations of Fe(II) from 1.3 to 190 μM and O_2_ from <3 to 123 μM within the mats (Table 1). Mariana mats had shallow O_2_ gradients, while at Loihi, O_2_ was undetectable (<3 μM) at 1 cm below the mat surface. These Fe(II) and O_2_ conditions favor biotic Fe oxidation (10). At Mariana, total dissolved Fe was depleted by 49% to 74% in our low-temperature mats relative to the conservative mixing of the local high-temperature zero-Mg endmember (Fig. S2), which suggests that a substantial amount of Fe is being oxidized and precipitated within these mats.

**TABLE 1:**
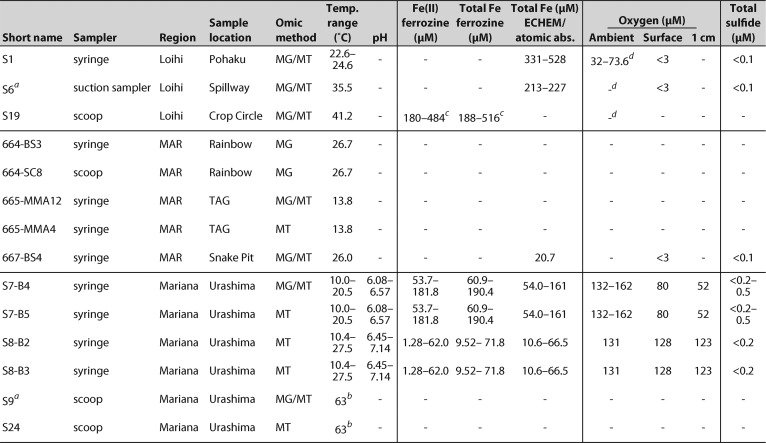
Summary of key geochemistry for each sample^*e*^

a 16S and MG samples taken from pre-T0 time point.

b Temperature ∼0.5 m within mat.

c Data from vent fluids collected by major sampler.

d Ambient O_2_ concentrations at Loihi Seamount approximately 60 μM.

e Symbols and abbreviations: −, no data; MAR, Mid-Atlantic Ridge; MG, metagenome; MT, metatranscriptome.

10.1128/mSystems.00553-19.1 TEXT S1 Supplemental methods, text, and references. Download Text S1, PDF file, 0.8 MB. Copyright © 2020 McAllister et al. 2020 McAllister et al. This content is distributed under the terms of the Creative Commons Attribution 4.0 International license.

10.1128/mSystems.00553-19.2 FIG S1 Photographs of sampling locations for samples chosen for metagenome and metatranscriptome sequencing from Loihi Seamount (A), Mid-Atlantic Ridge (B), and Mariana Backarc (C). Images on the left show context for the specific sampling location, which is marked by an arrowhead and depicted on the right. Download FIG S1, PDF file, 2.4 MB. Copyright © 2020 McAllister et al. 2020 McAllister et al. This content is distributed under the terms of the Creative Commons Attribution 4.0 International license.

10.1128/mSystems.00553-19.3 FIG S2 Urashima Vent Field (Mariana) geochemistry, plotted to highlight mixing of a single endmember with seawater. Mn and Si are conservative, or unreactive, during mixing (A). Total dissolved Fe is depleted in low-temperature fluids compared to conservative mixing (B; enlarged in panel C). This is particularly pronounced within the Golden Horn Chimney Fe mats. Baltan is a high-temperature vent in the Urashima Vent Field. Saipanda is another low-temperature vent that was not sampled for this study. Download FIG S2, PDF file, 0.7 MB. Copyright © 2020 McAllister et al. 2020 McAllister et al. This content is distributed under the terms of the Creative Commons Attribution 4.0 International license.

10.1128/mSystems.00553-19.9 TABLE S1 Sample names, origin, and type for this study. Download Table S1, XLSX file, 0.05 MB. Copyright © 2020 McAllister et al. 2020 McAllister et al. This content is distributed under the terms of the Creative Commons Attribution 4.0 International license.

### *Zetaproteobacteria* abundance and diversity.

We initially assessed *Zetaproteobacteria* abundance and diversity using a 16S rRNA gene survey (Fig. S3). Fe mat communities at all sites hosted abundant *Zetaproteobacteria* populations, from 16.4 to 95.9% of the total bacterial community at Loihi, 10.7 to 31.3% at the Mid-Atlantic Ridge, and 37.1 to 79.9% at Mariana. Many samples were dominated by the *Zetaproteobacteria*, notably sample S1 (96% *Zetaproteobacteria*), a centimeter-scale sample of actively growing Fe mat surface. In addition to *Zetaproteobacteria*, the mats hosted variable flanking microbial communities that differed between the three sites (Fig. S3 and S4) but were similar to previous studies (9, 22, 23, 28, 29). Overall, the relatively simple, *Zetaproteobacteria*-rich composition of these marine Fe mats makes them good systems for studying neutrophilic Fe oxidation mechanisms.

10.1128/mSystems.00553-19.4 FIG S3 PacBio 16S rRNA gene survey of *Bacteria* (A) and *Zetaproteobacteria* (B) microbial communities from Fe mats at Loihi Seamount, the Mid-Atlantic Ridge, and Mariana Backarc. The abundance of *Zetaproteobacteria* is highlighted (A). Blue asterisks denote samples chosen for metagenomics. Red asterisks denote samples chosen for metatranscriptomics. Numbers at the bottom of the bar charts denote the number of total 16S rRNA gene sequences sampled. Sample short name and Fe mat type are also given. Download FIG S3, PDF file, 0.7 MB. Copyright © 2020 McAllister et al. 2020 McAllister et al. This content is distributed under the terms of the Creative Commons Attribution 4.0 International license.

10.1128/mSystems.00553-19.5 FIG S4 Comparison of 16S rRNA gene, metagenome, and metatranscriptome relative abundance for the microbial communities at Loihi Seamount (A), the Mid-Atlantic Ridge (B), and Mariana Backarc (C). 16S rRNA gene plots represent the bacterial population only. The relative abundance of the *Zetaproteobacteria* is tracked for samples from the same Fe mat location and/or for MT samples mapped to the same metagenomes. Asterisks show MT samples that were mapped to a reference MG from a different sample. Download FIG S4, PDF file, 1.6 MB. Copyright © 2020 McAllister et al. 2020 McAllister et al. This content is distributed under the terms of the Creative Commons Attribution 4.0 International license.

**FIG 1:**
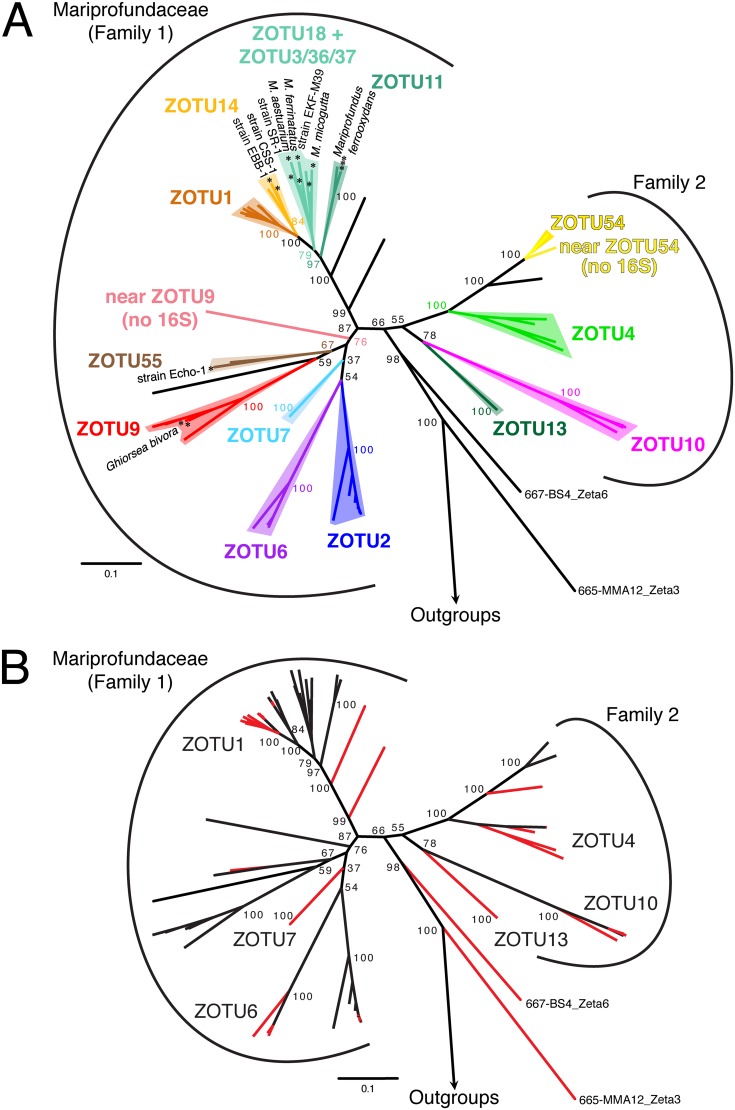
*Zetaproteobacteria* concatenated ribosomal protein reference maximum likelihood tree (100 bootstraps) showing the most commonly sampled ZOTUs (A) and highlighting genomes produced by this study (B). (A) All isolates of the *Zetaproteobacteria* are marked with an asterisk. Deep-branching genomes 665-MMA12_Zeta3 and 667-BS4_Zeta6 were classified as *Zetaproteobacteria*, though they are more deeply rooted than any prior lineage. (B) Genomes produced by this study are highlighted in red. Six ZOTUs that lacked sufficient depth for comparative genomics prior to this study are labeled.

**FIG 2:**
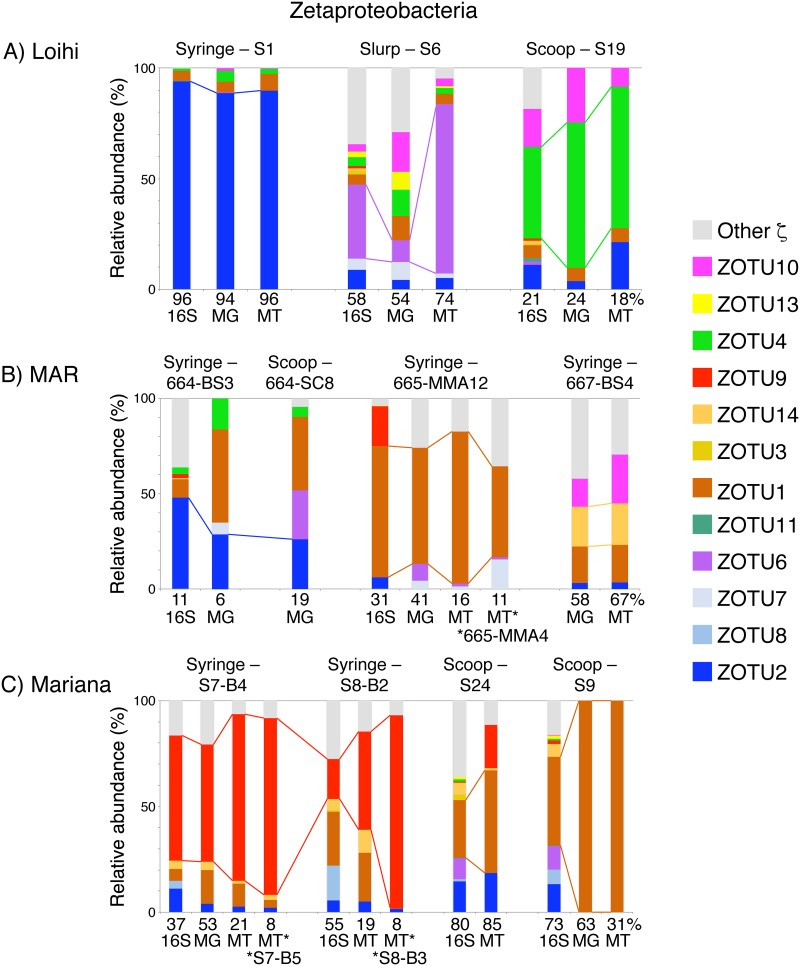
Comparison of 16S rRNA gene, metagenome (MG), and metatranscriptome (MT) relative abundance for the *Zetaproteobacteria* from various mats at Loihi Seamount (A), the Mid-Atlantic Ridge (B), and Mariana Backarc (C). The relative abundance of the most abundant *Zetaproteobacteria* operational taxonomic unit (ZOTU) by 16S rRNA gene is tracked across similar Fe mat samples from the same region. Asterisks denote MTs from different samples that were mapped to the indicated MG. Percentages are shown at the bottom of each bar graph to indicate the relative proportion of *Zetaproteobacteria* in each sample (see Fig. S4).

We used the 16S rRNA gene community profiling results to choose metagenomic (MG) and metatranscriptomic (MT) samples, aiming to recover high-abundance and diverse *Zetaproteobacteria* to produce high-quality genomes with sufficient MT read depth (Fig. S3). We recovered 126 total high-quality MAGs from our samples (>70% complete, <10% redundant) (see Tables S4 and S5 at https://doi.org/10.6084/m9.figshare.c.4646336) along with 79 improved MAGs by reanalyzing a Loihi metagenomic data set from the work of Fullerton et al. (9) (Text S1; see also Table S5 at https://doi.org/10.6084/m9.figshare.c.4646336). Of these, 53 MAGs belonged to the *Zetaproteobacteria* (selected genomes in Table S2), which were compared to a collection of published high-quality genomes (Text S1). MAGs from this study improve the representation of nine different *Zetaproteobacteria* operational taxonomic units (ZOTUs) spanning the *Zetaproteobacteria* phylogenetic tree by providing 2 to 13 additional high-quality MAGs for each of these ZOTUs (Fig. 1; Table S2). Many of these ZOTUs previously had poor genome representation (labeled ZOTUs in Fig. 1B). These diverse ZOTUs were abundant and active within our Fe mats (abundance by 16S rRNA gene and MG; activity by MT) (Fig. 2). MAG relative abundance generally matched relative activity, with the exception of MAG S6_Zeta1 (ZOTU6), which had higher activity than expected, likely in response to the shipboard incubation conditions. By substantially improving *Zetaproteobacteria* genome representation and pairing this with metatranscriptomes, we are poised to investigate genetic commonalities and diversity across the *Zetaproteobacteria*, particularly of the Fe oxidation mechanism.

10.1128/mSystems.00553-19.10 TABLE S2 *Zetaproteobacteria* genomes used in comparative genomics, concatenated ribosomal protein phylogenetic tree, and gene expression estimates. Download Table S2, XLSX file, 0.02 MB. Copyright © 2020 McAllister et al. 2020 McAllister et al. This content is distributed under the terms of the Creative Commons Attribution 4.0 International license.

### Phylogeny of the putative Fe oxidase Cyc2.

**FIG 3:**
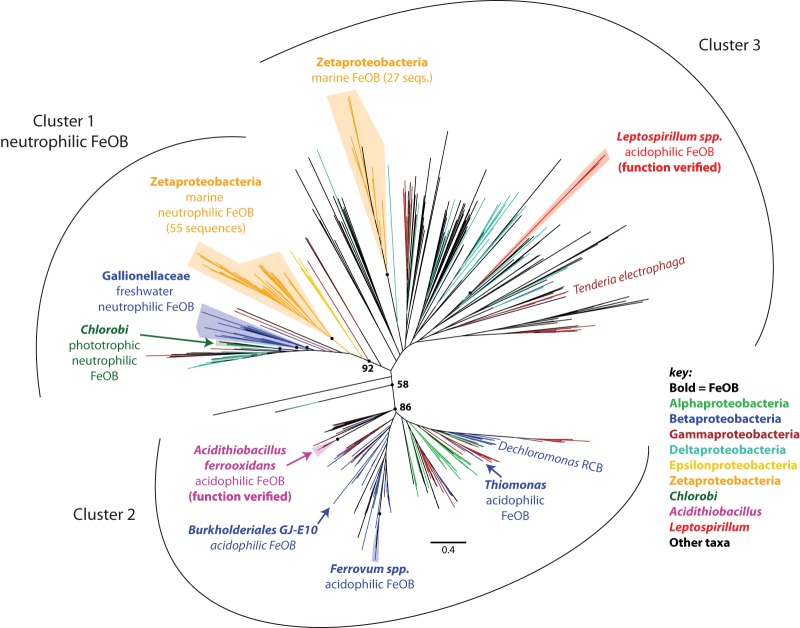
Cyc2 maximum likelihood phylogenetic tree (100 bootstraps), showing three distinct clusters. All groups of Fe-oxidizing bacteria are labeled, in addition to the electrode-oxidizing Tenderia electrophaga. *Zetaproteobacteria* and *Gallionellaceae* cluster with other neutrophilic Fe oxidizers in Cluster 1 (92% cluster support). Fe oxidation has been demonstrated for Cyc2 from Cluster 2 Acidithiobacillus ferrooxidans and Cluster 3 Leptospirillum sp. Unlabeled circles at nodes correspond to the following bootstrap values in parentheses: *Zetaproteobacteria* Cluster 1 (97%), *Gallionellaceae* (87%/63%), *Chlorobi* (99%), Acidithiobacillus ferrooxidans (100%), *Ferrovum* spp. (100%), *Zetaproteobacteria* Cluster 3 (99%), and *Leptospirillum* spp. (100%).

The key component of the proposed neutrophilic Fe oxidation pathway is Cyc2, which has been shown to oxidize Fe(II) in acidophiles. Our preliminary analyses showed that some *Zetaproteobacteria* genomes have multiple *cyc2* copies that were not closely related. To investigate these, we developed a comprehensive Cyc2 phylogeny. This phylogeny includes sequences from terrestrial to marine and circumneutral to acidic environments, as well as both known Fe oxidizers and organisms not known to oxidize Fe (Fig. 3; see Fig. S8 for detailed tree with sequence names at https://doi.org/10.6084/m9.figshare.c.4646336). Cyc2 sequences form three clusters, but the Fe-oxidizing function has been verified only for the Cluster 2 Acidithiobacillus ferrooxidans Cyc2 (11) and the Cluster 3 Cyc2 homolog Cyt_572_ of *Leptospirillum* sp. (12). However, most of the neutrophilic Fe oxidizers fall within Cluster 1, a well-supported group (93% bootstrap) that is largely comprised of the *Zetaproteobacteria*, *Gallionellaceae*, and Chlorobium ferrooxidans. This strongly suggests that Cluster 1 Cyc2s share a function.

### Putative Fe oxidation pathway distribution revealed by comparative genomics of the *Zetaproteobacteria*.

**FIG 4:**
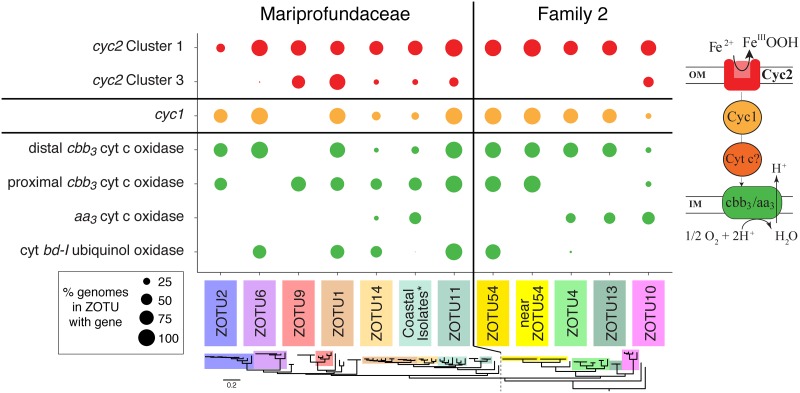
Dot plot showing the distribution of genes from the putative Fe oxidation pathway between major ZOTUs. Each dot represents the percentage of genomes in the ZOTU which possess the gene of interest. Genes are colored by their relative position within the core putative Fe oxidation pathway, shown at right. ZOTUs are ordered by the reference ribosomal protein tree (bottom), separated into the two families of the *Zetaproteobacteria*. The coastal isolate group (see asterisk) includes isolates Mariprofundus aestuarium CP-5, Mariprofundus ferrinatatus CP-8, *Mariprofundus* sp. strain SR1, *Mariprofundus* sp. strain EKF-M39, and Mariprofundus micogutta ET2.

Our Cyc2 phylogeny shows that *Zetaproteobacteria* possess Cyc2 from both Clusters 1 and 3. All ZOTUs possess a Cluster 1 *cyc2* gene; with our new genomes, this includes four additional ZOTUs that are now known to possess *cyc2* (ZOTUs 1, 7, 13, and 14; Fig. 4). In contrast, fewer ZOTUs have the Cluster 3 *cyc2* gene, and 65% of genomes with Cluster 3 *cyc2* (*n *= 15) also have Cluster 1 *cyc2*. This suggests that both Cyc2 types have a use in the *Zetaproteobacteria*, though it is unknown how Cluster 1 and 3 Cyc2s may differ in function. ZOTU2 is unusual in that only 3 of the 10 genomes appear to have *cyc2*, though this may be due to assembly issues specific to ZOTU2 (Text S1). In any case, the presence of *cyc2* in all *Zetaproteobacteria* OTUs suggests its centrality to these neutrophilic Fe oxidizers.

**FIG 5:**
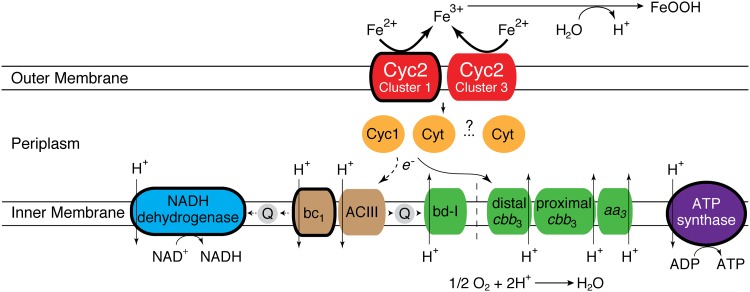
Proposed Fe oxidation pathway model showing variation in the genetic capability of all *Zetaproteobacteria*. Components that are conserved in all *Zetaproteobacteria* are outlined with a thick line. Components of the pathway from the same module have the same color.

In addition to *cyc2*, other proposed genes for the Fe oxidation pathway were also widely distributed in *Zetaproteobacteria* genomes. Homologs of *cyc1* were present in the genomes of all ZOTUs except ZOTU9 (Fig. 4); *cyc1* encodes a diheme *c*-type cytochrome thought to be a periplasmic electron carrier in the *A. ferrooxidans* Fe oxidation pathway (11). ZOTU9, which includes the Fe- and H_2_-oxidizing *Ghiorsea* spp. (30), must use another periplasmic electron carrier. Indeed, many other putative periplasmic cytochromes can be found in *Zetaproteobacteria* genomes (see below). Cyc1 or another electron carrier likely passes electrons to a terminal oxidase or to complex I via complex III (reverse electron transport). Genes for the *bc*_1_ complex were found in all ZOTUs, whereas we found alternative complex III (*ACIII*) genes in only a few *Zetaproteobacteria*, primarily in ZOTU11 and Family 2 (ZOTUs 4, 10, and 13). We found three types of aerobic terminal oxidases: (i) *cbb*_3_-type cytochrome *c* oxidase, (ii) *aa*_3_-type cytochrome *c* oxidase, and (iii) cytochrome *bd-*I ubiquinol oxidase. Further, two distinct forms of the *cbb*_3_-type cytochrome *c* oxidase were found, clustering in the proximal and distal *cbb*_3_ subtrees defined by Ducluzeau et al. (31) (Fig. S5). All ZOTUs possess genes for one or more of these terminal oxidases, suggesting that all *Zetaproteobacteria* are aerobic Fe oxidizers (Fig. 4). Taken together, these findings allow us to update the neutrophilic Fe oxidation pathway model (Fig. 5).

10.1128/mSystems.00553-19.6 FIG S5 Maximum likelihood phylogenetic tree of CcoN, showing that the *Zetaproteobacteria* possess two forms of *cbb*_3_-type cytochrome *c* oxidase: proximal and distal. Only the distal version is found within the conserved cassette discovered by Field et al. (E. K. Field, A. Sczyrba, A. E. Lyman, C. C. Harris, et al., ISME J 9:857–870, 2015, https://doi.org/10.1038/ismej.2014.183). Download FIG S5, PDF file, 0.7 MB. Copyright © 2020 McAllister et al. 2020 McAllister et al. This content is distributed under the terms of the Creative Commons Attribution 4.0 International license.

### *In situ* expression of the putative Fe oxidation pathway.

Our next step was to determine whether the putative Fe oxidation pathway genes are expressed in the environment; high expression would lend support for the model. *In situ* expression from six unique ZOTUs in 10 different samples (total of 21 observations) shows that *cyc2* genes from Cluster 1 are highly expressed in all *Zetaproteobacteria* and samples, ranging from 3.0× to 555× baseline constitutive gene expression (Table 2). Cluster 1 *cyc2* was frequently the highest-expressed gene in the genome, particularly in the *Mariprofundaceae* (Family 1). Interestingly, *cyc2* expression levels differed between *Zetaproteobacteria* Families 1 and 2, though expression was still high in all *Zetaproteobacteria*. The *cyc1* and *cbb*_3_-type terminal oxidase genes are expressed up to 17.3× and 56.6× constitutive gene expression, respectively. On average, this places the *cyc1* and terminal oxidase genes in the 73.4 and 75.4 percentile range in *Zetaproteobacteria* metatranscriptomic expression, respectively (Fig. S6). This gene expression is consistent with protein expression levels of M. ferrooxydans PV-1, where the corresponding proteins were expressed at or above the 87th percentile (14). In combination, our data suggest that genes in the core model Fe oxidation pathway are highly expressed *in situ* by diverse *Zetaproteobacteria* under various environmental conditions.

**TABLE 2:**
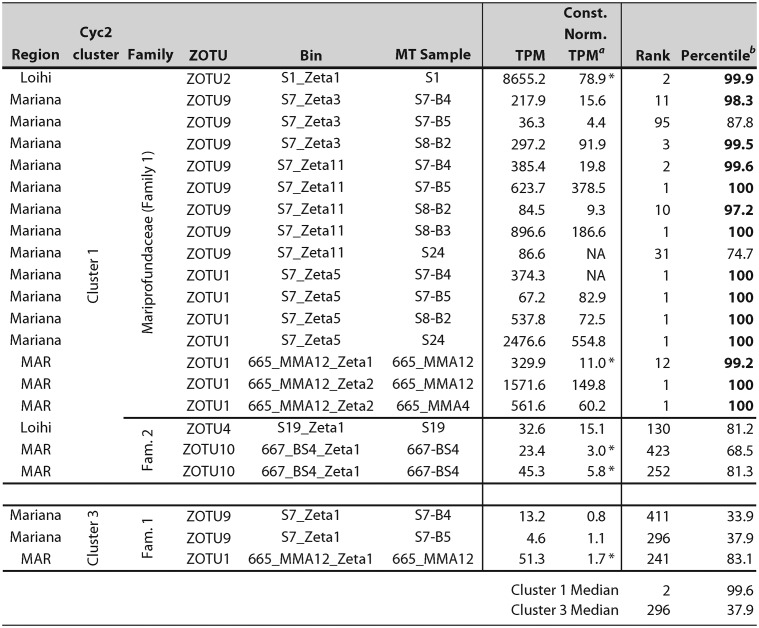
Expression and relative importance of *cyc2* genes from *in situ* samples

a Const. Norm. TPM, constitutive normalized TPM. Asterisks denote constitutive normalized TPM from bins with sufficient read depth (see Table S7 at https://doi.org/10.6084/m9.figshare.c.4646336). NA, constitutive normalized expression cannot be calculated.

b High *cyc2* expression above the 90th percentile indicated in boldface.

10.1128/mSystems.00553-19.7 FIG S6 Heat map showing the percentile expression for key genes in the Fe oxidation pathway, including genes thought to be involved in electron transport from Fe(II) to O_2_ and from Fe(II) to the quinone pool for reverse electron transport (RET). Download FIG S6, PDF file, 0.7 MB. Copyright © 2020 McAllister et al. 2020 McAllister et al. This content is distributed under the terms of the Creative Commons Attribution 4.0 International license.

Variation in gene expression may help us resolve which modules are most commonly used during Fe oxidation in the environment. For example, expression of complex III module genes further supports the importance of *bc*_1_ over ACIII for reverse electron transport. Average expression of *bc*_1_ was 6.6× constitutive expression, while *ACIII* expression was much lower at 0.6×. In ZOTUs with both complexes, *bc*_1_ genes were expressed 1.5× to 18.6× higher than the *ACIII* complex. The limited distribution of *ACIII* in only a few *Zetaproteobacteria* lineages, despite our deep sampling with near-complete genomes, combined with its low expression suggests that ACIII is not required for Fe oxidation under the sampled conditions.

Comparison of relative *in situ* expression may also help identify genes that may be involved as intermediate electron carriers, particularly in ZOTU9, which lacks *cyc1*. We identified at least 14 different putative periplasmic *c*-type cytochrome genes (PCs) with high expression (>90th percentile) in one or more *Zetaproteobacteria* genomes (see Table S6A at https://doi.org/10.6084/m9.figshare.c.4646336). Some of these genes (*cyc1*, *PC12*, *PC61*, *PC16*, and *PC38*) were more highly expressed in some genomes than *cyc2* in the *Mariprofundaceae*, and all were found in at least one genome where they were more highly expressed than *cyc1*. Interestingly, these putative periplasmic cytochromes were found and expressed at different levels in different *Zetaproteobacteria* lineages, with some unique to a single ZOTU (e.g., *PC73* in ZOTU2) and most found in several ZOTUs. The cytochromes *c* previously found on a conserved cassette identified in *Zetaproteobacteria* isolates and single amplified genomes (SAGs) (including *cyc1*, *PC2*, and *PC3*) (15) are most highly expressed in genomes from ZOTUs 1 and 2. These results help us narrow the list of potential electron carriers in the *Zetaproteobacteria*.

### Expression of Fe oxidation pathway genes in Fe-amended mat incubations.

To link gene expression more specifically with Fe oxidation, we added Fe(II) to mat samples and analyzed metatranscriptomic responses over time. We performed shipboard incubations at Loihi Seamount and Mariana, using fresh Fe mats, live and killed, while monitoring Fe oxidation. Microbes within the mat were actively oxidizing Fe(II) faster than abiotic processes, with the pseudo-first-order Fe oxidation rate constants 3.7× (Loihi) and 5.3× (Mariana) higher in live samples than azide-killed ones (Fig. S7). These results show that we stimulated biotic Fe oxidation, which should lead to increased expression of Fe oxidation genes.

10.1128/mSystems.00553-19.8 FIG S7 Plot of Fe(II) addition experiment results from Loihi (A) and Mariana (B) Fe mats, showing a higher living (orange; total) than killed (black; abiotic-only) Fe oxidation rate. Fe(II) was added to dormant Fe mat samples at 0 min. Pseudo-first-order rate constants were calculated from the log-linear best fit from each experimental condition. Download FIG S7, PDF file, 0.7 MB. Copyright © 2020 McAllister et al. 2020 McAllister et al. This content is distributed under the terms of the Creative Commons Attribution 4.0 International license.

**FIG 6:**
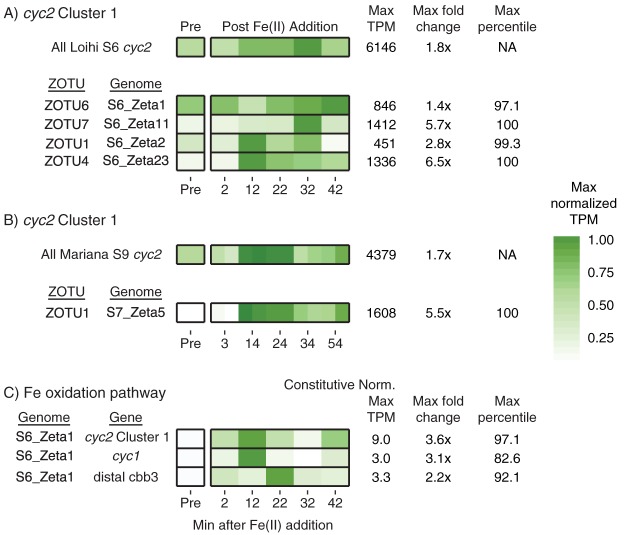
Normalized TPM expression changes for *cyc2* and other Fe oxidation pathway genes in the Fe(II) amendment experiments, showing increases after Fe(II) addition. (A and B) TPM expression changes are shown for all Cluster 1 *cyc2* and for *cyc2* from specific *Zetaproteobacteria* MAGs at Loihi (A) and Mariana (B) (duplicates shown). NA, not applicable. (C) Constitutive normalized expression changes shown for the Fe oxidation pathway in MAG S6_Zeta1. S9 MT data mapped to the S7 MG for expression estimates of MAGs in panel B. TPM values were maximally normalized to emphasize peak expression.

As with the *in situ* samples, *cyc2* was highly expressed in the *Zetaproteobacteria* during the time series experiments, reaching a maximum of 97.1st to 100th percentile in the four most active *Zetaproteobacteria* lineages. After Fe(II) was added, there was an increase in total *cyc2* expression (sum of all *cyc2* genes), as well as *cyc2* expression by each individual MAG (Fig. 6A and B). Expression increased at different rates for each ZOTU, with some peaking earlier and others peaking at the end of the experiment. Expression of *cyc2* increased less for the most abundant *Zetaproteobacteria* (e.g., S6_Zeta1), which already had high expression of *cyc2* prior to Fe(II) amendment. Less-abundant *Zetaproteobacteria* (S6_Zeta11/S6_Zeta23) were also expressing *cyc2* prior to Fe(II) amendment but had a much larger change in expression (5.7× to 6.5×), reached their maximum quickly, and maintained a higher expression over the course of the experiment. Overall, while the degree and timing of response differed, all *Zetaproteobacteria* increased their *cyc2* expression in response to Fe(II) amendment.

Other genes in the Fe oxidation pathway also generally followed this trend, with *cyc1* expression in 4 of 5 genomes and *cbb*_3_-type terminal oxidase genes in 2 of 4 genomes also increasing after Fe(II) addition. However, low read recruitment depth led to substantial noise. To correct for this noise, we normalized expression using six constitutively expressed genes (Text S1; also see Table S7 at https://doi.org/10.6084/m9.figshare.c.4646336) and focused on expression patterns in S6_Zeta1, which had high read depth (Fig. 6C). Constitutive normalized expression of S6_Zeta1 shows a similar pattern of *cyc2* expression change over the time series compared to expression patterns prior to constitutive normalization, with a maximum increase of 3.6× after Fe(II) amendment. The genes encoding Cyc1 and the *cbb*_3_-type terminal oxidase also increased after Fe(II) addition, reaching a maximum fold change of 1.5× to 3×. This trend was also observed for 7 of 8 putative periplasmic cytochromes more highly expressed than *cyc1*, increasing 2.1× to 4.5× over the course of the experiment (see Table S6B at https://doi.org/10.6084/m9.figshare.c.4646336). These results suggest that the Fe(II) amendment increased the expression of many genes thought to be in the Fe oxidation pathway.

If the *Zetaproteobacteria* represented by the S6_Zeta1 genome is an autotrophic Fe oxidizer, Fe(II) amendment should stimulate genes for carbon fixation, central metabolism, and growth. Like *cyc2* expression, genes for central metabolic pathways, including the tricarboxylic acid (TCA) cycle, increased in expression 2.0× to 3.4× over 1 h in S6_Zeta1 from ZOTU6 (see Fig. S9 at https://doi.org/10.6084/m9.figshare.c.4646336). Similarly, expression of genes for glycogen synthesis increased 1.8× in the first 2 min after Fe(II) addition. Carbon fixation genes increased 1.9× in the first 12 min. Some of the highest fold changes after Fe(II) amendment were seen in genes related to proper protein folding (molecular chaperones *groEL*, *groES*, and *dnaK*) and membrane protein quality control (*htpX-*type protease) (32). For example, *groEL* increased 119× after Fe(II) addition. Though these gene responses may correspond with shock to the cell after Fe(II) amendment, these genes were also highly expressed under *in situ* conditions in S1_Zeta1, which suggests that they may be necessary for promoting active Fe oxidation in the environment. Together, these data suggest that Fe(II) amendment stimulated genes for both Fe oxidation and growth.

## DISCUSSION

The main objective of this study was to assess the Cyc2-based Fe oxidation pathway in neutrophilic Fe-oxidizing bacteria (FeOB), using environmental metagenomics and metatranscriptomics of marine Fe mats. This work contributes 53 new nearly complete *Zetaproteobacteria* genomes paired with expression profiles, both *in situ* and from incubations, along with a comprehensive Cyc2 phylogeny. Using these, we have characterized the distribution and usage of the model Fe oxidation pathway across the full range of known *Zetaproteobacteria* in Fe mats at three geographically distinct venting regions. The emerging pattern shows that the pathway as a whole is highly expressed, with increased expression in all Fe pathway genes following Fe(II) amendment. The *cyc2* gene is among the highest expressed and is the only gene in the pathway shared by all *Zetaproteobacteria*, suggesting that it plays a key role in Fe oxidation.

### Assessing the Fe oxidation pathway model through *Zetaproteobacteria* comparative genomics.

In this study, we assessed the current model for Fe oxidation by comparing genomes representing the full diversity of the *Zetaproteobacteria* (Fig. 1B). Since the *Zetaproteobacteria* are thought to be an entire class of Fe-oxidizing bacteria, all genomes should have an Fe-oxidizing pathway, which may be conserved. Our results show that overall, the basic model of neutrophilic Fe oxidation in the *Zetaproteobacteria* holds (Fig. 4). All *Zetaproteobacteria* lineages, including novel ones presented in this study, possess genes encoding the putative Fe oxidase Cyc2, an intermediate electron carrier, and a terminal oxidase. However, our survey shows that each of these components can have multiple versions, suggesting that the pathway contains interchangeable modules, as depicted in our updated model (Fig. 5). Across all *Zetaproteobacteria*, there are two types of Cyc2, multiple potential periplasmic cytochrome electron carriers, and four terminal oxidases. The variations are likely linked with specific adaptations related to niche, with some genomes possessing multiple versions of certain components, perhaps to span multiple niches (33). Within each ZOTU, individual genomes possessed pathway gene variations consistent with the ZOTU as a whole, even though some genomes were missing genes that a majority of others in the ZOTU possessed. These false negatives could result from incomplete MAGs, which is why we focused on ZOTUs. Accounting for modularity and genome variability within ZOTUs, comparative genomics has confirmed that this Fe oxidation pathway is common to all *Zetaproteobacteria* lineages.

### Support for *cyc2* as an Fe oxidation gene in neutrophilic Fe oxidizers.

Cyc2 homologs in neutrophilic FeOB are commonly referred to as “putative Fe oxidases” based on homology to the functionally characterized *A. ferrooxidans* Cyc2, though sequence similarity is low. Indeed, our phylogenetic analysis shows that Cyc2 sequences are highly diverse, and most of the neutrophilic FeOB Cyc2 homologs fall into Cluster 1 (Fig. 3). This cluster forms a distinct group from the clusters containing biochemically characterized Fe oxidases: Cyc2 from *A. ferrooxidans* (Cluster 2) and Cyt_572_ from *Leptospirillum* sp. (Cluster 3). Although none of the Cluster 1 Cyc2s have been biochemically characterized, the high bootstrap support suggests a common function, and the prevalence of sequences from Fe-oxidizing isolates strongly suggests involvement in Fe oxidation.

Another clue to function lies in the expression of *cyc2* in Fe mat environments, where Fe oxidation by the *Zetaproteobacteria* is required for carbon fixation and growth. Genes central to fitness are often highly expressed (34, 35). We measured *cyc2* expression in five different active Fe mats from three different hydrothermal vent fields and confirmed that *cyc2* is frequently the highest-expressed gene. This is true in diverse *Zetaproteobacteria* lineages (Table 2), suggesting that the pathway is important to *Zetaproteobacteria* fitness in many different environments. The level of *cyc2* expression in the *Zetaproteobacteria* is consistent with the high expression in Fe-oxidizing isolates of *Acidithiobacillus* sp. (often above microarray detection limits) and *Ferrovum* sp. (8× above average expression) (36–38). In the environment, the neutrophilic *Gallionellaceae* have been shown to express *cyc2* highly, up to the 100th percentile in an Fe-rich aquifer (39). Together with the expression of *cyc1* and terminal oxidase genes, this shows that the putative Fe oxidation pathway is consistently expressed under Fe-oxidizing conditions. The especially high expression of *cyc2* across various Fe-oxidizing taxa and Fe-oxidizing environments supports its role in Fe oxidation in both acidophiles and neutrophiles.

To link *cyc2* to Fe oxidation, we followed its expression when Fe(II) was added to Fe mat samples. In separate experiments at Loihi and Mariana, Fe(II) amendment resulted in both active biotic Fe oxidation (see Fig. S7 in the supplemental material) and increased *cyc2* gene expression. This increase in expression was found not only for the whole sample but also in every *Zetaproteobacteria* genome detected within these samples (Fig. 6). Although there was variation in the timing and magnitude of the response, which may be lineage specific, the fact that expression increased in all *Zetaproteobacteria* suggests that *cyc2* expression is stimulated by the presence of Fe(II). The Fe(II) amendment also resulted in increases in carbon fixation and central metabolism gene expression, suggesting a link between *cyc2* expression, neutrophilic Fe oxidation, and growth (see Fig. S9 at https://doi.org/10.6084/m9.figshare.c.4646336).

### Can the *cyc2* gene be used as a marker for Fe oxidation?

Unlike many other energy metabolisms, neutrophilic Fe oxidation is challenging to track in the environment due to the lack of an isotopic signature and difficulties distinguishing biotic from abiotic Fe oxides. Until recently, there have not been any candidates for a widely applicable Fe oxidation genetic marker; instead, it seemed that there were many different potential Fe oxidases, with various levels of functional verification (e.g., references 6, 7, and 40). Our work adds to the mounting evidence that Cyc2 is an Fe oxidase. The *cyc2* gene is widely distributed across many Fe-oxidizing lineages, with homologs in acidophiles and neutrophiles. Specifically for neutrophiles, *cyc2* is common across the well-studied neutrophilic chemolithotrophs *Gallionellaceae* and *Zetaproteobacteria*. As we have sequenced more of these neutrophilic FeOB genomes, this association has held true (9, 13, 15, 21, 41). However, our Cyc2 phylogeny has identified a substantial number of cultured and uncultured organisms that have not yet been shown or tested to be capable of Fe oxidation, work that could bolster confidence. In all, the *cyc2* gene is a promising genomic marker of the capacity for Fe oxidation across many different Fe-oxidizing lineages, including neutrophiles.

Not only is *cyc2* common to all well-established neutrophilic Fe oxidizers, it is also highly expressed in environments where neutrophilic Fe oxidizers predominate (this study and reference 39). This opens the possibility of *cyc2* expression levels as an indicator of microbial Fe oxidation activity. Indeed, when we stimulated *Zetaproteobacteria* Fe oxidation in incubations, expression of *cyc2* increased along with an increase in carbon fixation and central metabolism genes. This is consistent with Fe-oxidation-fueled chemolithoautotrophic growth, and so relative *cyc2* expression levels can correspond to increases in Fe oxidation activity. However, our results suggest that *cyc2* expression levels may not be easily related to Fe oxidation activity in the environment. All *Zetaproteobacteria* in our samples were expressing *cyc2* prior to Fe(II) amendment, when there was no detectable dissolved Fe(II). This could represent baseline expression by obligate Fe oxidizers, which always need to be prepared for Fe oxidation. In this case, relative changes in *cyc2* expression would remain more informative for activity. Alternatively, *cyc2* expression before Fe(II) amendment could result from cryptic cycling of Fe between Fe oxidizers and reducers (42). Such cryptic cycling would make developing a genetic marker for activity even more important for tracking Fe oxidation activity. Because of these potential complications, further transcriptomics experiments should focus on isolates or microcosms without Fe reducers. In combination with our results, such experiments will help us understand how to use *cyc2* expression levels to interpret Fe oxidation activity in the environment.

### Conclusions.

Using paired metagenomes and metatranscriptomes from the *Zetaproteobacteria*, we have been able to demonstrate that the Cyc2-based neutrophilic Fe oxidation pathway is widespread and highly expressed in the environment, validating the environmental importance of the pathway. We have shown that the Cluster 1 *cyc2* gene, conserved in the *Zetaproteobacteria* and other neutrophilic Fe oxidizers, is highly expressed in multiple Fe mat environments and is stimulated by Fe(II) addition, suggesting it may be regulated. This makes *cyc2* an excellent marker of Fe oxidation capability and may allow us to detect and monitor the activity of Fe oxidizers in the environment. However, to correlate expression with activity, further efforts should focus on testing the regulation of *cyc2* in diverse organisms and simple communities. The phylogeny of Cyc2 shows at least three distinct clusters, with some neutrophilic Fe oxidizers possessing multiple copies (e.g., Clusters 1 and 3 in the *Zetaproteobacteria*). This may be akin to an example in Pseudomonas aeruginosa, which has multiple *cbb*_3_-type cytochrome *c* oxidases that are optimized for high and low O_2_ concentrations and for resistance to respiratory inhibitors cyanide and nitrite and thus allow growth under different conditions (43). If Cyc2 variants are similarly optimized, they may enable Fe oxidation under various conditions, a hypothesis that could be tested by independently monitoring *cyc2* from different clusters in diverse habitats and growth conditions. Without a marker of activity, the roles of neutrophilic Fe oxidizers have been virtually invisible outside model Fe-oxidizing environments, like Fe microbial mats. By applying our findings to other environments, we can start to reveal how Fe-oxidizing microbes drive key biogeochemical cycles in the varied marine and freshwater habitats where they thrive.

## MATERIALS AND METHODS

### Biological sample collection.

Samples were collected from various vent fields on three separate cruises to the Mid-Atlantic Ridge (2012), Loihi Seamount (2013), and the Mariana Backarc (2014) (see Table S1 in the supplemental material). To preserve *in situ* expression, 18 samples were collected using devices half-filled with 2× RNAlater (Invitrogen, Carlsbad, CA, USA). Samples collected using a syringe sampler device (44) provided ∼10 to 30 ml of mat material representing a discrete microbial population, as opposed to 2 liters (scoop) or >5 liters (suction sample) of bulk sample. After settling for a few hours at 4°C, the overlying supernatant was removed and samples were stored at −80°C.

### Geochemical measurements and sampling.

At the Mid-Atlantic Ridge and Loihi Seamount, geochemistry was measured *in situ* using cyclic voltammetry (ECHEM), as described in the work of MacDonald et al. (45) (MAR) and Chan et al. (26) (Loihi). The detection limits were 3 μM O_2_, 7 to 10 μM Fe^2+^, and 0.1 μM sulfide (45, 46).

At Mariana, geochemistry samples were collected using the hydrothermal fluid and particle sampler (HFPS) (47) or the microbial mat sampler (44). The HFPS pulls fluid through a titanium inlet nozzle at 1 to 4 liters/min. The fluid flows through a continuously flushed titanium and Teflon manifold and is diverted into sample containers or to a SeaBird (Bellevue, WA) SBE 63 oxygen sensor and an AMT (Rostock, Germany) deep-sea glass pH electrode. In extremely low-outflow vent environments, seawater will be entrained in the HFPS and dilute the *in situ* fluid. We collected temperature, pH, and O_2_ concentrations for ambient water, at the surface of the chimney, and with the nozzle inserted into the microbial mat. HFPS chemistry results represent the fluid composition at the measured temperature, including any entrained seawater that occurs during sampling. The microbial mat sampler has a much lower intake rate (<0.2 liter min^−1^) and is better able to capture chemical microenvironments. The microbial mat sampler was equipped with 0.22-μm inline filtering and a check valve for chemical analysis. Fe(II) and total Fe concentrations were assayed using the ferrozine method with 40 mM sulfamic acid to stabilize Fe(II) (48, 49); the detection limit was estimated at 0.12 μM Fe(II). Samples recovered with the HFPS were processed as described previously (47) and analyzed shipboard for pH by glass electrode and on shore for total dissolved iron by atomic absorption at National Oceanic and Atmospheric Administration/Pacific Marine Environmental Laboratory (NOAA/PMEL) and by inductively coupled plasma mass spectrometry (ICPMS) at the University of Washington Department of Civil Engineering.

### Fe(II) amendment experiments.

Shipboard Fe(II) amendment experiments were conducted on bulk mat samples from Loihi (J2-677-SSyellow) and Mariana (J2-801-SC8). Samples were transported to the ship after 2 h (Loihi) and 11 h (Mariana) of remotely operated vehicle (ROV) operations and allowed to settle at 4°C for 1 h prior to removing the majority of the supernatant and starting the experiment. One sample was taken immediately prior to Fe(II) amendment [pre-Fe(II) addition]. Water bath temperature and initial Fe(II) amendment concentration were set to mimic environmental conditions. Fe oxidation pseudo-first-order rate constants (*k*_1_) were calculated using a log-linear fitted trend line.

At Loihi Seamount, Fe mat floc was added to two 250-ml vessels; one remained alive while the other was killed using 1 mM azide, which has been shown to interact with Fe(II), though not at this concentration and time interval (50). Both vessels were shaken by hand several times a minute in a 35°C water bath. To initiate the experiment, 100 μM FeCl_2_ was added. After this, starting after 2 min and subsequently at 10-min intervals, samples from each vessel were removed for Fe(II) and total Fe measurements by the ferrozine method (48). Concurrently, 30 ml from the living vessel was mixed 1:1 with 2× RNAlater (Invitrogen). This mixture was held at 4°C for a few hours prior to freezing at −80°C.

At Mariana, Fe mat material was sparged with a 2% O_2_ gas mix (pH 5.9/6.2 before/after sparge). Each time point (*n *= 5) and treatment (duplicate living and 3 mM azide killed) had its own 125-ml reaction vessel with 30 ml mat material. In addition to a pre-Fe(II) addition sample, one sample was taken at the end which did not experience any Fe(II) addition. Both of these nonamended samples had low Fe(II) concentrations (below detection [BD] and 0.3 μM, respectively). Each reaction vessel was amended with 333 μM FeCl_2_ and suspended in a 28°C water bath with frequent mixing by hand. Starting after 4 to 6 min and subsequently at 10-min intervals, one vessel was sacrificed at each time point, for sampling for Fe(II), total Fe, and pH and mixing of 25 ml of material 1:1 with 2× RNAlater.

### DNA and RNA extraction.

DNA samples were extracted using the FastDNA Spin kit for soil (MP Biomedicals, Santa Ana, CA, USA) according to the manufacturer’s instructions, except that 250 μl of a 0.5 mM sodium citrate solution (pH 5.8) was added prior to lysis. RNA samples were extracted using the NucleoSpin RNA kit (Macherey-Nagel, Bethlehem, PA, USA), with modifications detailed in Text S1. Prior to library preparation, an internal *in vitro*-transcribed RNA standard, pTXB1, was added (Text S1; see also Table S3 at https://doi.org/10.6084/m9.figshare.c.4646336). RNA extractions were used for metatranscriptome library preparation after nondegraded total RNA (visible 16S and 23S rRNA peaks) was confirmed by a fragment analyzer (2.2 to 9.3 RNA quality number [RQN]; median 5.7 RQN) (Advanced Analytical, Ankeny, IA, USA).

### 16S rRNA, metagenome, and metatranscriptome sequencing.

Microbial community composition was first estimated using a PacBio-based 16S rRNA gene survey, with SILVAngs used for taxonomic classification (see Text S1) (75). *Zetaproteobacteria* operational taxonomic units (ZOTUs) were classified from these 16S rRNA gene sequences using ZetaHunter (51). Samples were chosen for metagenomic (MG) and metatranscriptomic (MT) sequencing based on the microbial community composition and *Zetaproteobacteria* diversity. MG and MT libraries were prepared and sequenced at the University of Delaware Sequencing and Genotyping Center. Sequencing details are provided in Text S1.

### Metagenome assembly, binning, and annotation.

Raw sequence reads were trimmed to remove adaptors, poor-quality regions, and short sequences (Trimmomatic) (52), and paired reads were merged if overlapping (Flash) (53). Metagenome libraries were assembled from quality-controlled (QC’ed) reads using metaSPAdes v3.10, with read error correction disabled to improve recovery of real community genomic variation (54). Only contigs mapping ≥1× read coverage over 90% of their length were utilized in downstream analysis (∼92% remained).

Metagenome-assembled genomes (MAGs) were binned using four binning programs: MaxBin (55), MetaBAT (superspecific and very sensitive settings) (56), CONCOCT (57), and BinSanity (58). The resulting bins were combined and dereplicated using DAS Tool (59). Manual taxonomic and outlier (guanine-cytosine (GC) content/coverage) curation of bins was performed in ggkbase (https://ggkbase.berkeley.edu/), with additional curation performed using Anvi’o v3 (60). Finalized curated bins were tested for completeness and redundancy using CheckM (61) and classified using PhyloSift (62), and gene calling and SEED annotation were performed using RAST (63). RAST gene calls were used for Clusters of Orthologous Groups (COG) annotation within Anvi’o (60, 64), and Kyoto Encyclopedia of Genes and Genomes (KEGG) annotation was performed through BlastKOALA (65). Genes of interest (e.g., *cyc2*, *cyc1*, and terminal oxidases) were further manually curated based on evidence using NCBI BLASTp (66) against *Zetaproteobacteria* protein references. Gene annotation was assessed with maximum likelihood phylogenetic trees built from alignments using RAxML (67). The Cyc2 phylogenetic tree was constructed from an alignment of 634 unique Cyc2 protein sequences identified from NCBI and IMG databases using BLASTp (66, 68, 69). Additional information on the Cyc2 sequences and tree construction is provided in Text S1.

### RNA read recruitment and expression estimates.

Raw total RNA reads were quality controlled (see above) using Trimmomatic (average 99% of reads passed) (52). rRNA reads were removed using SortMeRNA (v2.1b) (70). The resulting non-rRNA reads, primarily mRNA, were used for subsequent recruitment for expression estimates. MT reads were recruited to the MG from the same sample, with the following exceptions: 665-MMA4 was recruited to 665-MMA12; S7_B5, S8_B2, S8_B3, S9, and S24 were recruited to S7_B4 MG. Reads were mapped using Bowtie 2, with default parameters (71).

To determine gene read recruitment, we used BEDTools to extract the read count from each gene coordinate region (72). We used three normalization methods for estimating gene expression: (i) transcripts per million (TPM), normalizing for sequencing effort and gene and read lengths (73); (ii) TPM values further normalized to the average expression of six constitutively expressed genes (*adk*, *gyrA*, *recA*, *rpoB*, *rpoC*, and *secA*) (74) to correct for changes in organism relative abundance (constitutive normalized expression); and (iii) TPM values normalized to the maximum expression for the time series for visual representation.

### Data accessibility.

High-quality full-length reads (20-pass minimum) from the PacBio 16S rRNA gene survey were submitted to GenBank (MK048478 to MK048944). Raw metagenome and metatranscriptome reads, as well as 5-pass-filtered PacBio 16S rRNA gene reads, were submitted to the NCBI SRA under BioProject accession PRJNA555820. Metagenome assemblies from this study and reassembled metagenome assemblies from the work of Fullerton et al. (9) were submitted to the JGI IMG database (sequence project IDs Gp0295814 to Gp0295821 and Gp0295823 [this study]; analysis project IDs Ga0256915 and Ga0257019 to Ga0257023 [Fullerton et al. {9}]). *Zetaproteobacteria* MAGs were also submitted to the JGI IMG database (see sequence project IDs listed above). Specific accession numbers per sample are shown in Table S4 at https://doi.org/10.6084/m9.figshare.c.4646336.
