# Supplementary material for: Validating the Cyc2 Neutrophilic Iron Oxidation Pathway Using Meta-omics of Zetaproteobacteria Iron Mats at Marine Hydrothermal Vents
Source: mSystems. 2020 Feb 18;5(1):e00553-19. doi: 10.1128/mSystems.00553-19 (PMC7029218; doi:10.1128/mSystems.00553-19)

A) Loihi Seamount (2013)

Sample S1
J2-674-BM1-C3
Pohaku (Mkr 57)
Syringe
RNALater

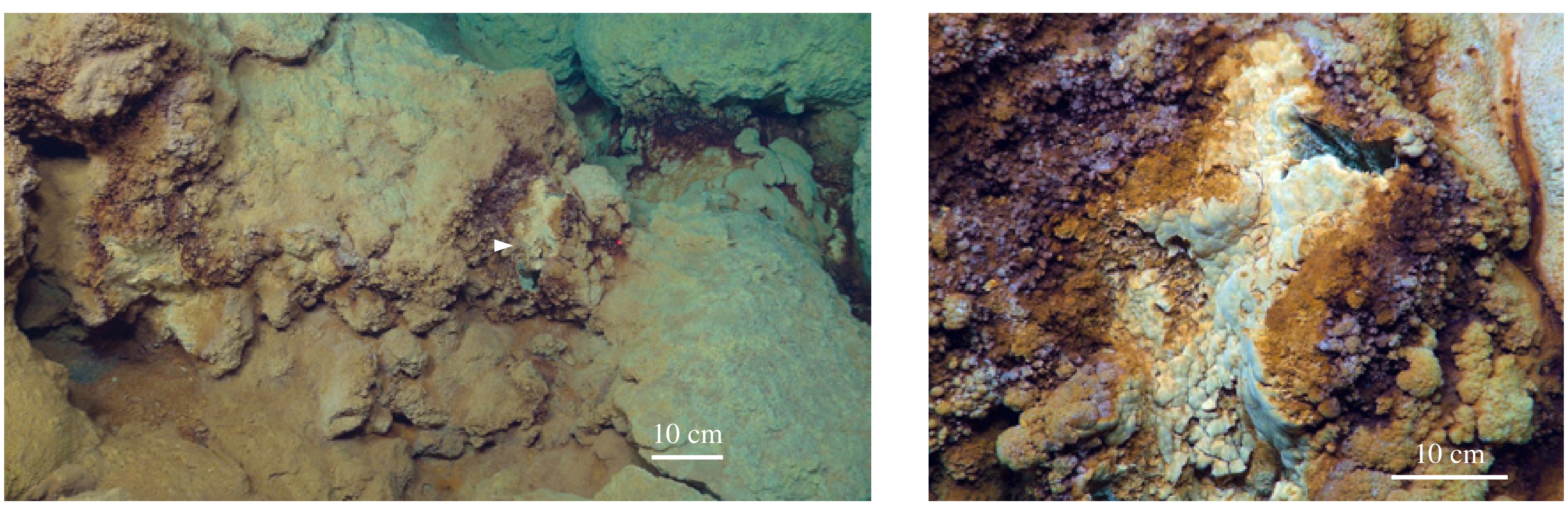

Sample S6
J2-677-SSyellow
Spillway (Mkr 34)
Suction sampler
Onboard experiment

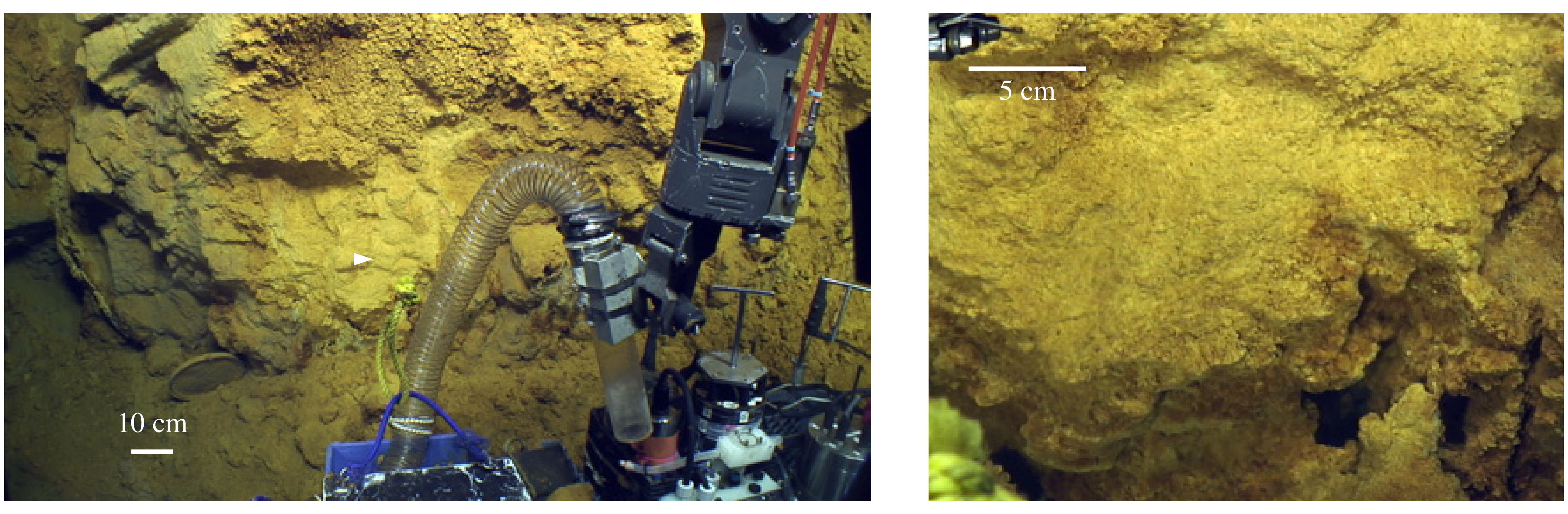

Sample S19
J2-675-SC9
Crop Circle (U Mkr 31)
Scoop
RNALater

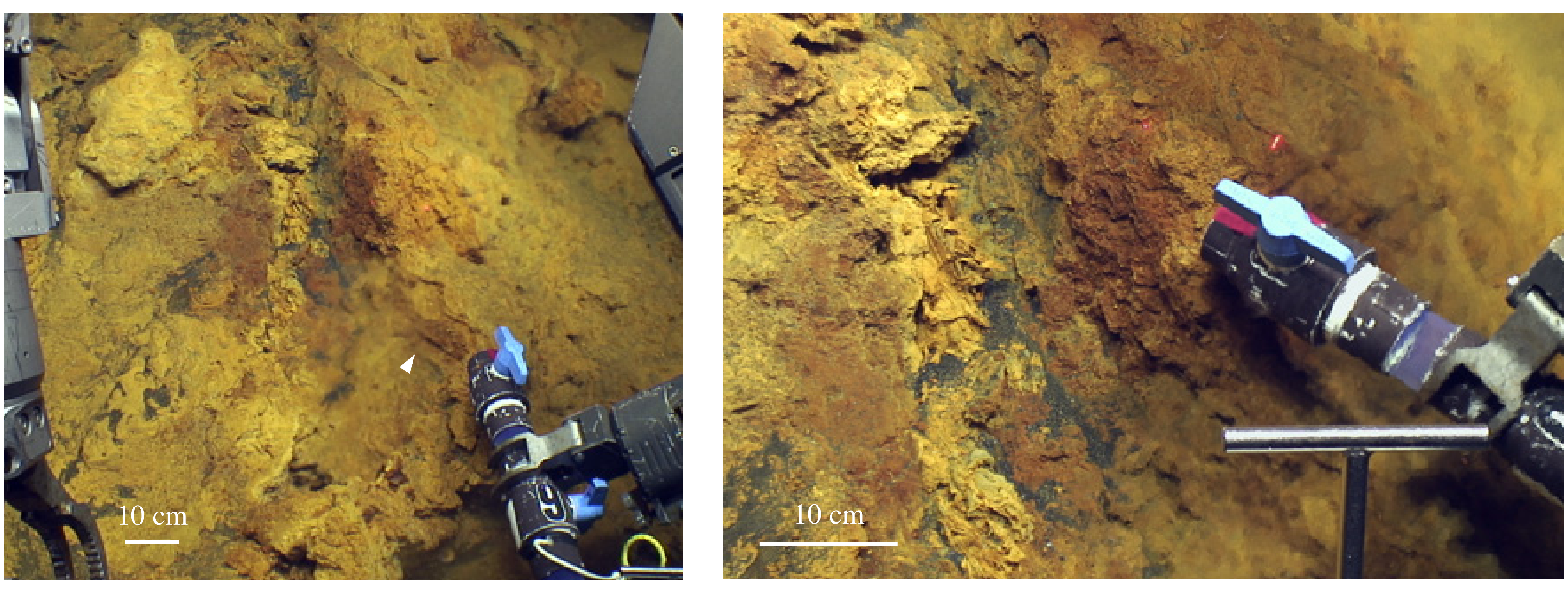

B) Mid-Atlantic Ridge (2012)

J2-664-BS3/
J2-664-SC8
Rainbow vent field
Syringe/Scoop
Untreated

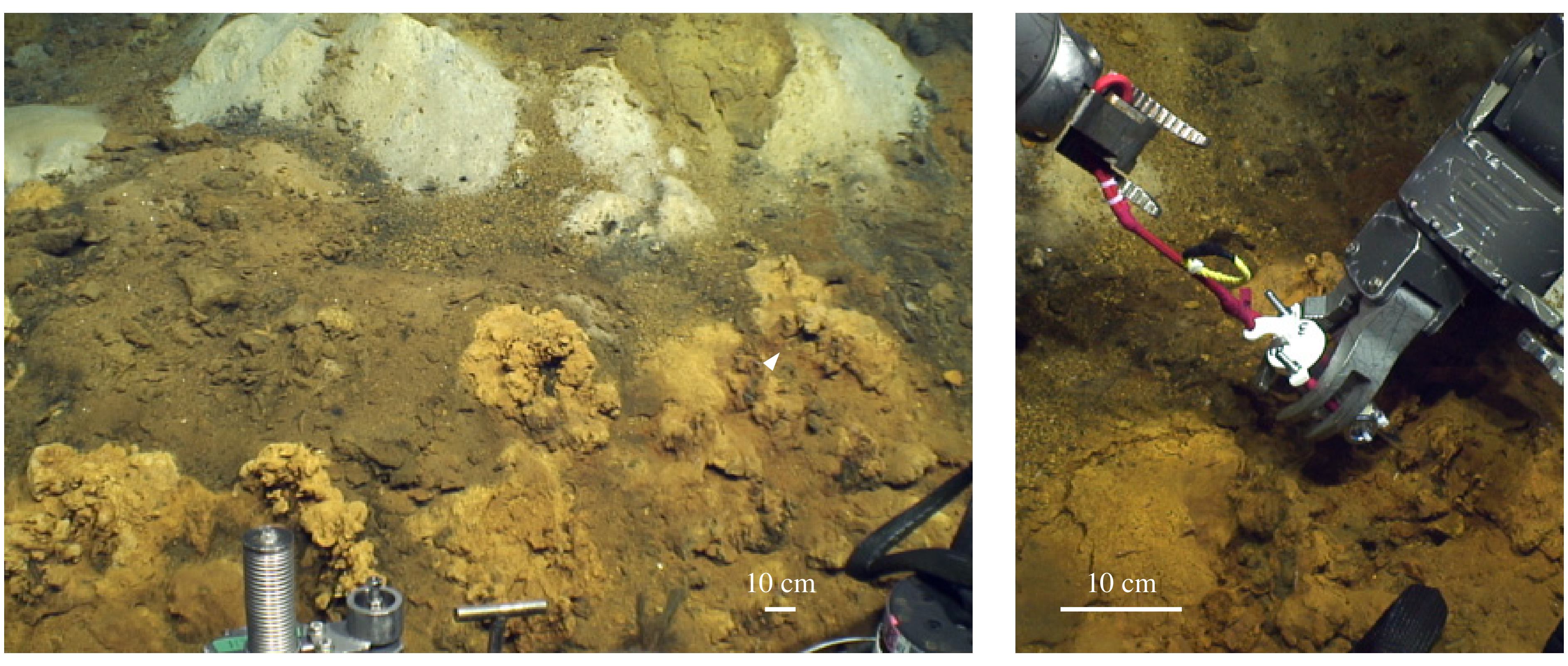

J2-665-MMA12
TAG vent field
Syringe
RNALater

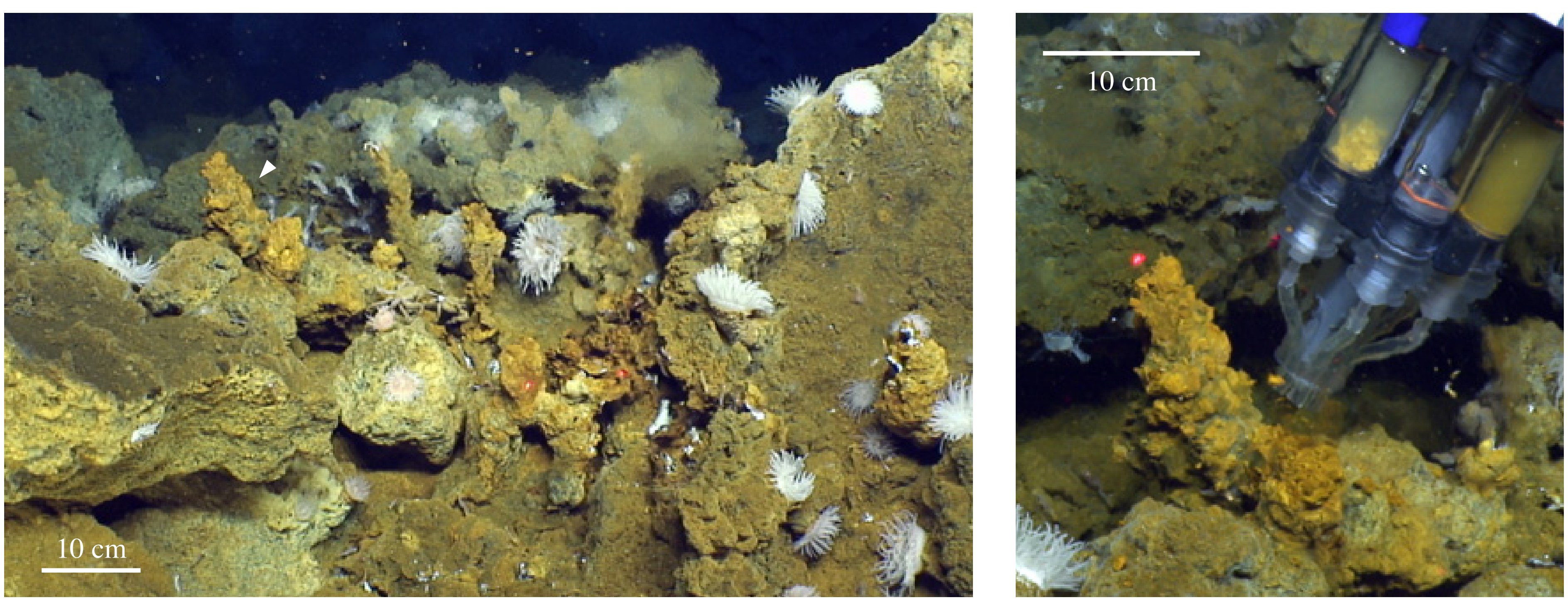

J2-667-BS4
Snake Pit vent field
Syringe
RNALater

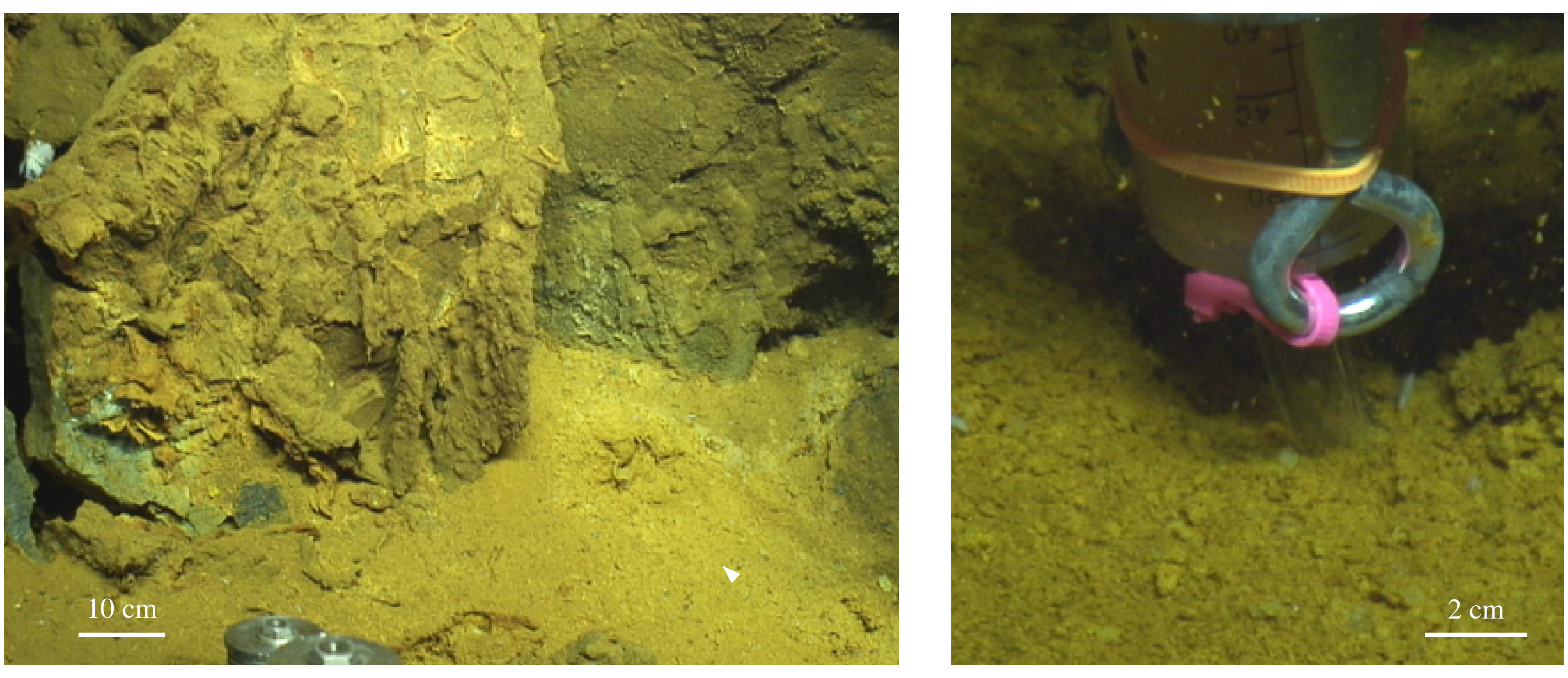

C) Mariana Backarc (2014)

Sample S7-B4/B5
J2-801-BM1-B4/B5
Urashima vent field
Golden Horn Chimney
Syringe
RNALater

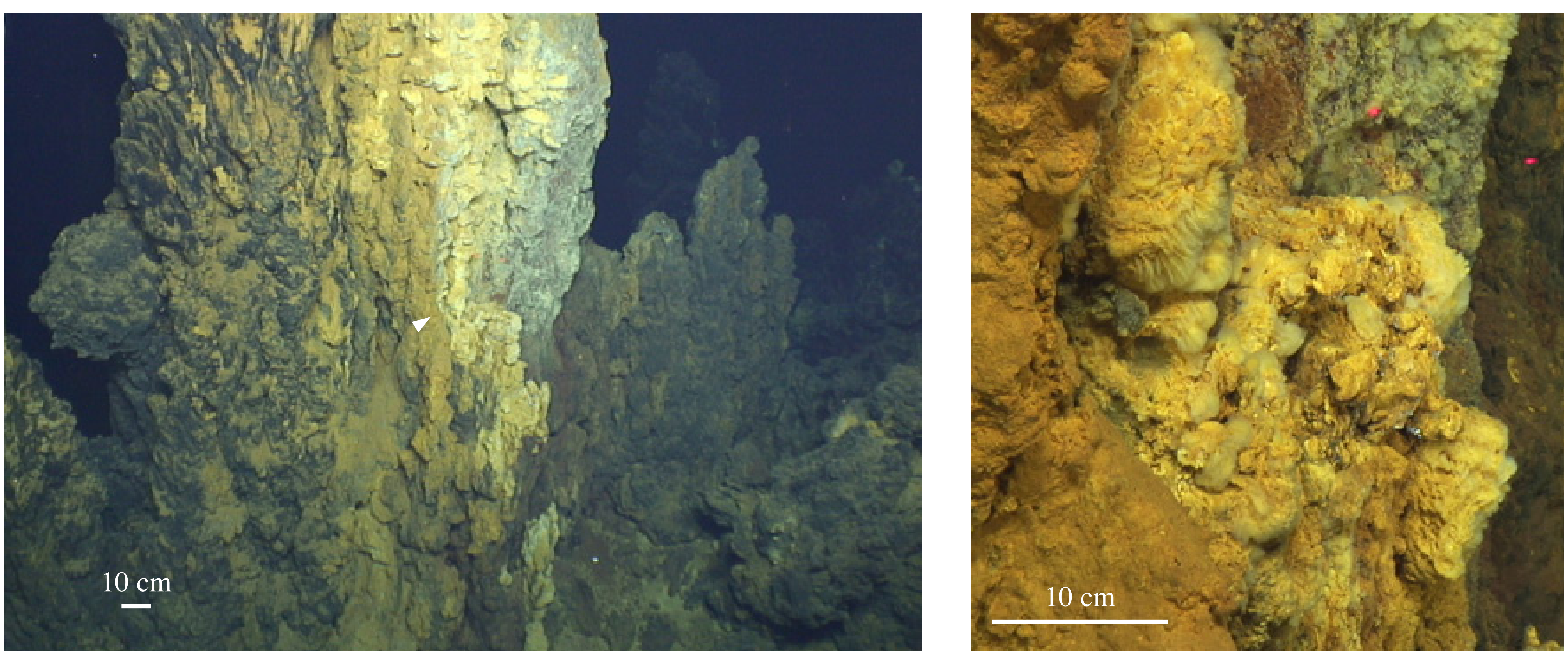

Sample S8-B2/B3
J2-801-BM1-B2/B3
Urashima vent field
Golden Horn Chimney
Syringe
RNALater

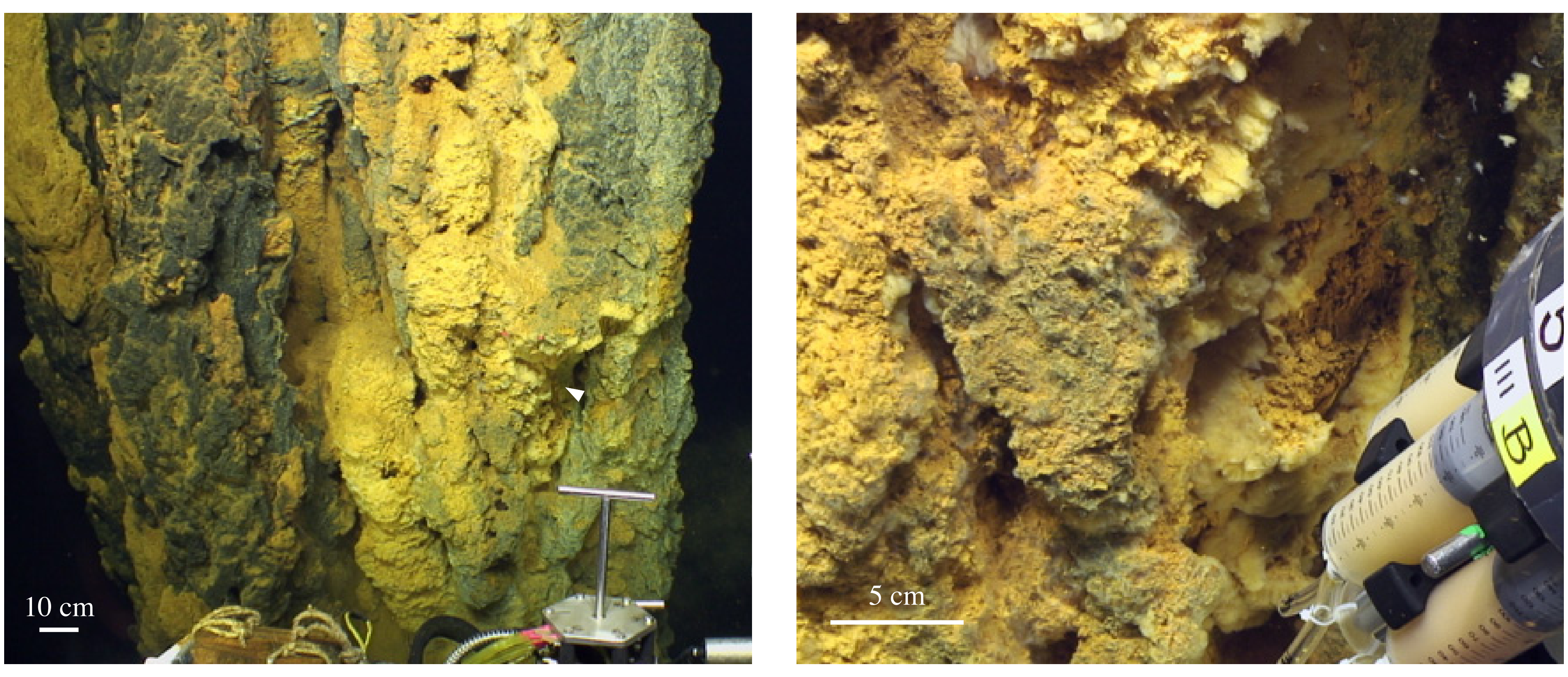

Sample S9
J2-801-SC8
Urashima vent field
Golden Horn Chimney
Scoop
Onboard experiment

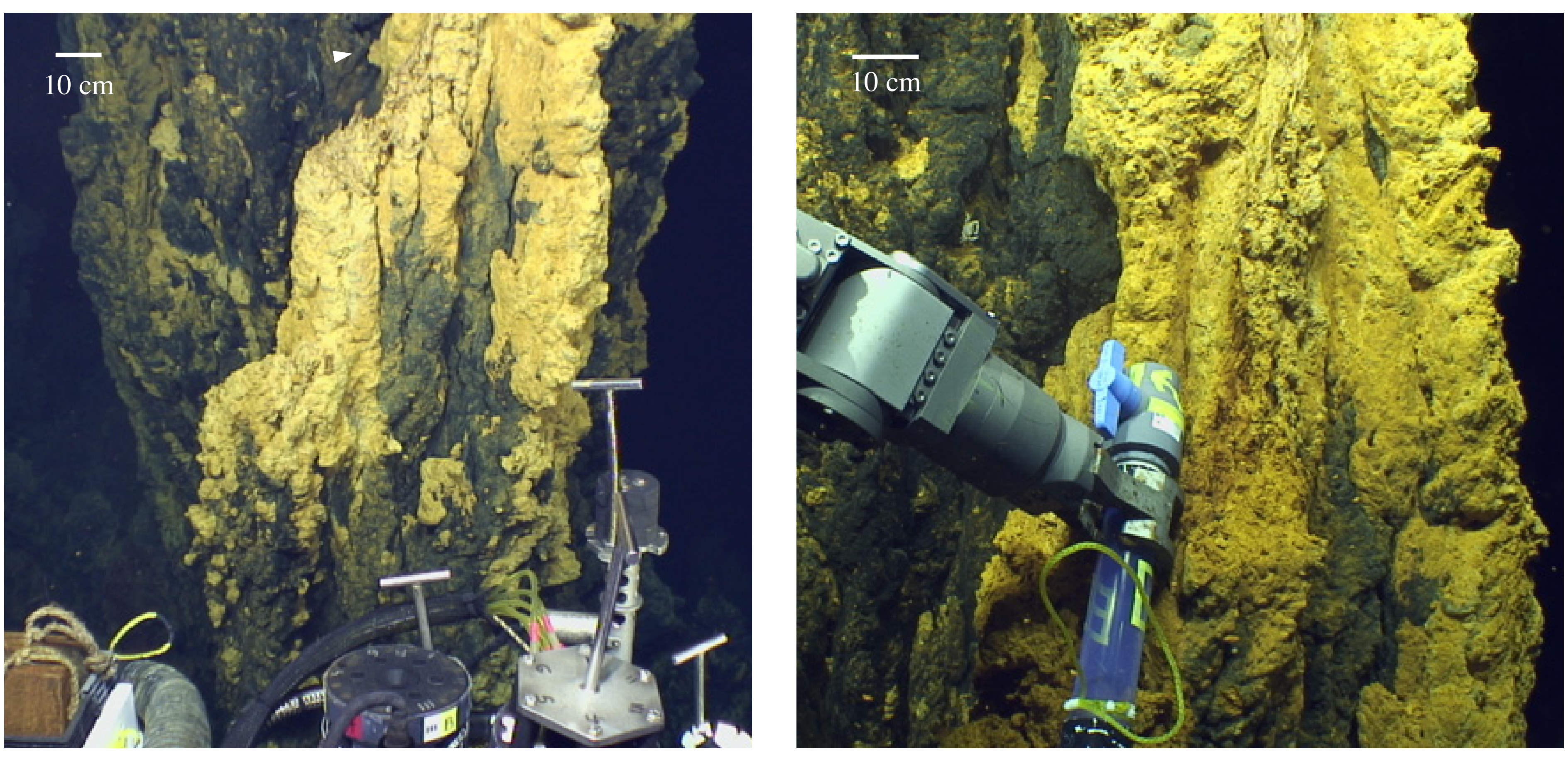

Supplement: FIG S1 [file mSystems.00553-19-sf001.pdf]
