# Supplementary figures and images for: Validating the Cyc2 Neutrophilic Iron Oxidation Pathway Using Meta-omics of Zetaproteobacteria Iron Mats at Marine Hydrothermal Vents
Source: mSystems. 2020 Feb 18;5(1):e00553-19. doi: 10.1128/mSystems.00553-19 (PMC7029218; doi:10.1128/mSystems.00553-19)

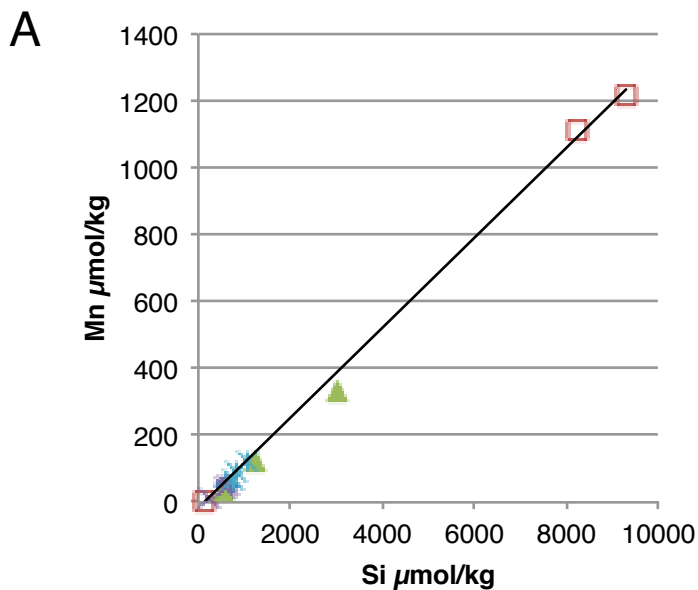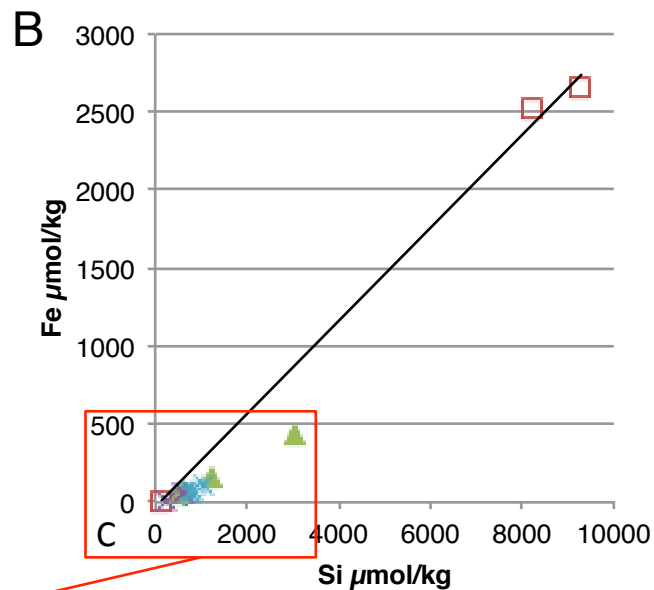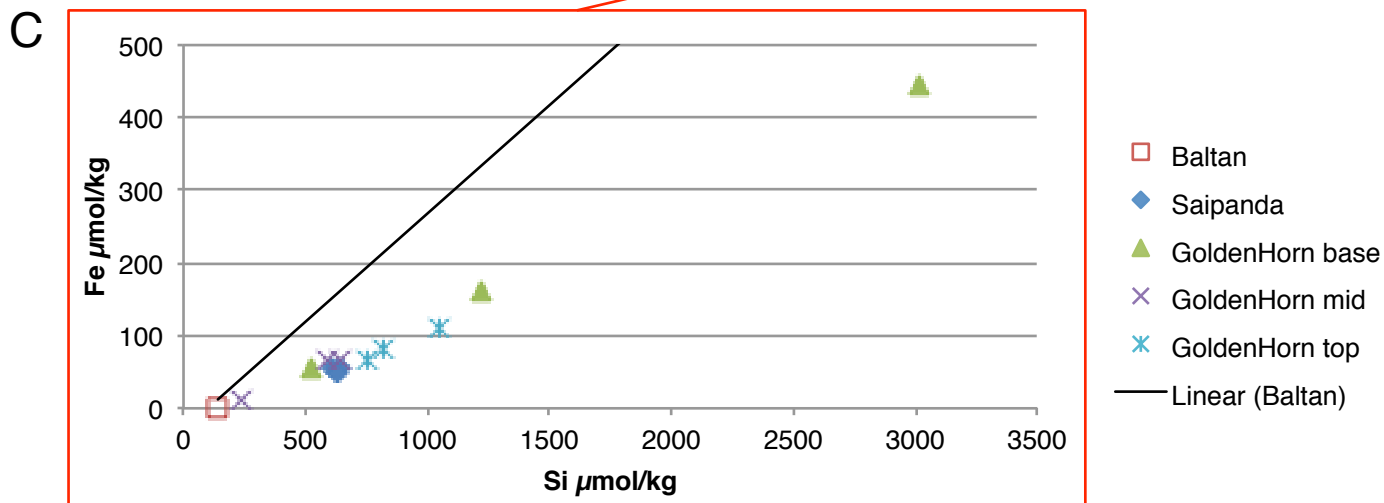

Supplement: FIG S2 [file mSystems.00553-19-sf002.pdf]

A

# Microbial Community

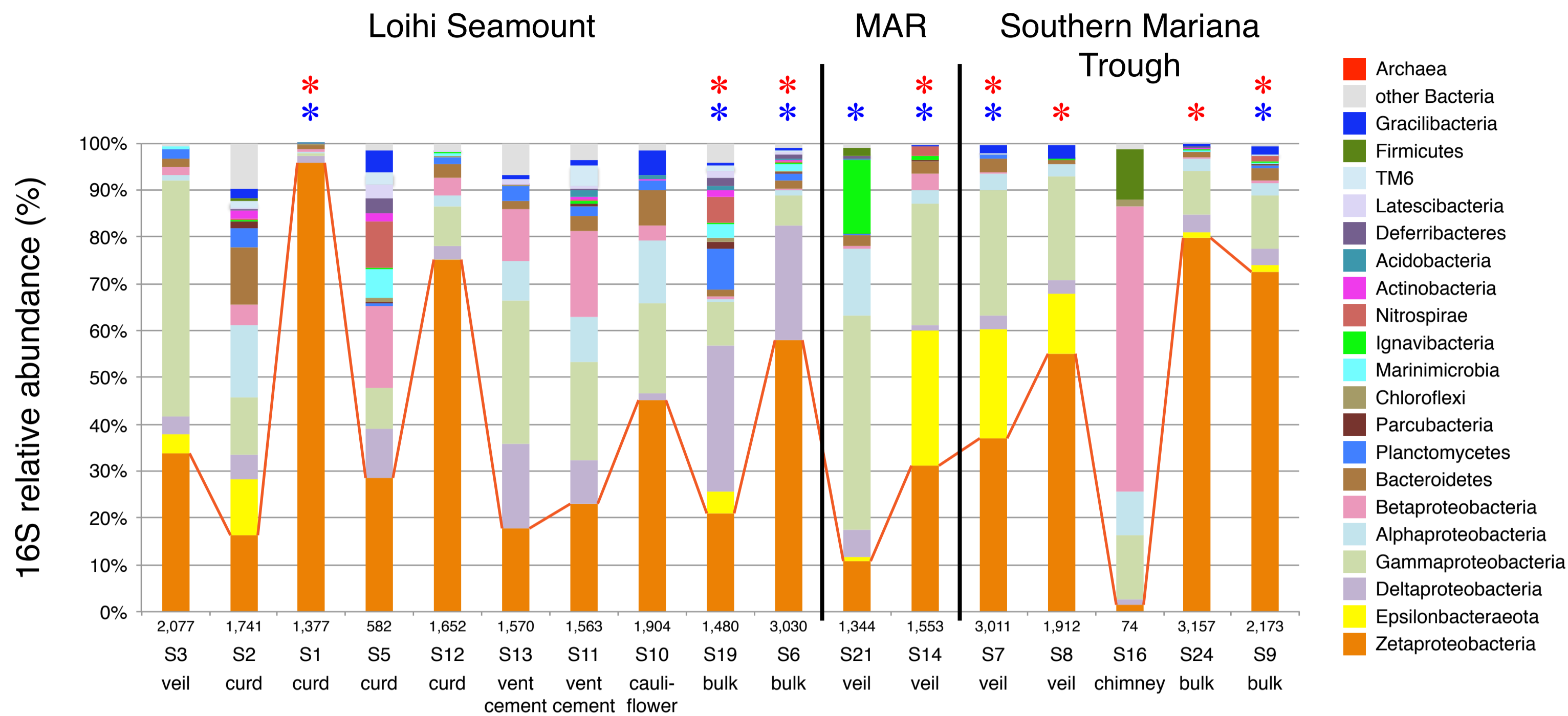

B

# Zetaproteobacteria

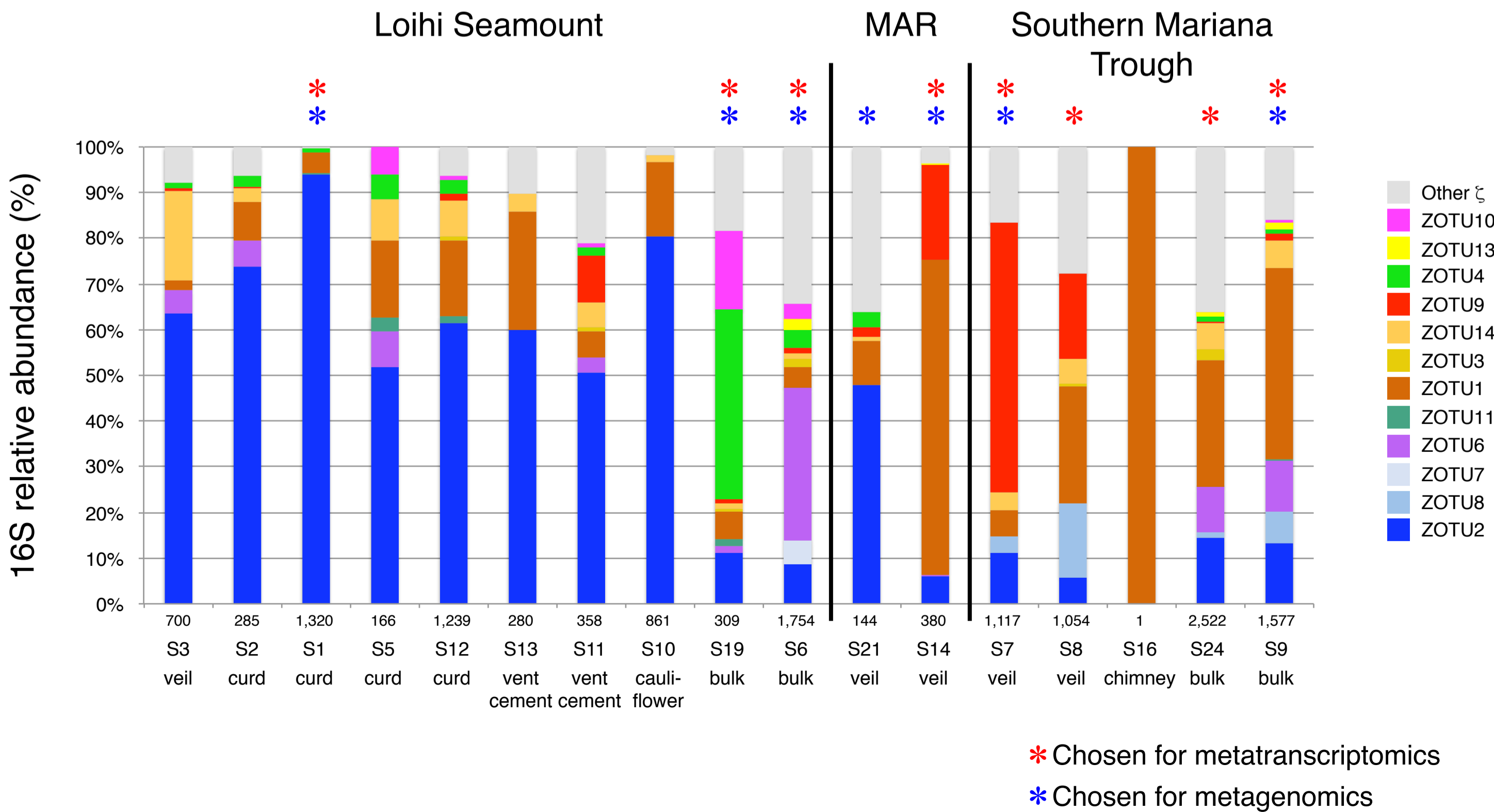

Supplement: FIG S3 [file mSystems.00553-19-sf003.pdf]

# Microbial Community

**A** Loihi

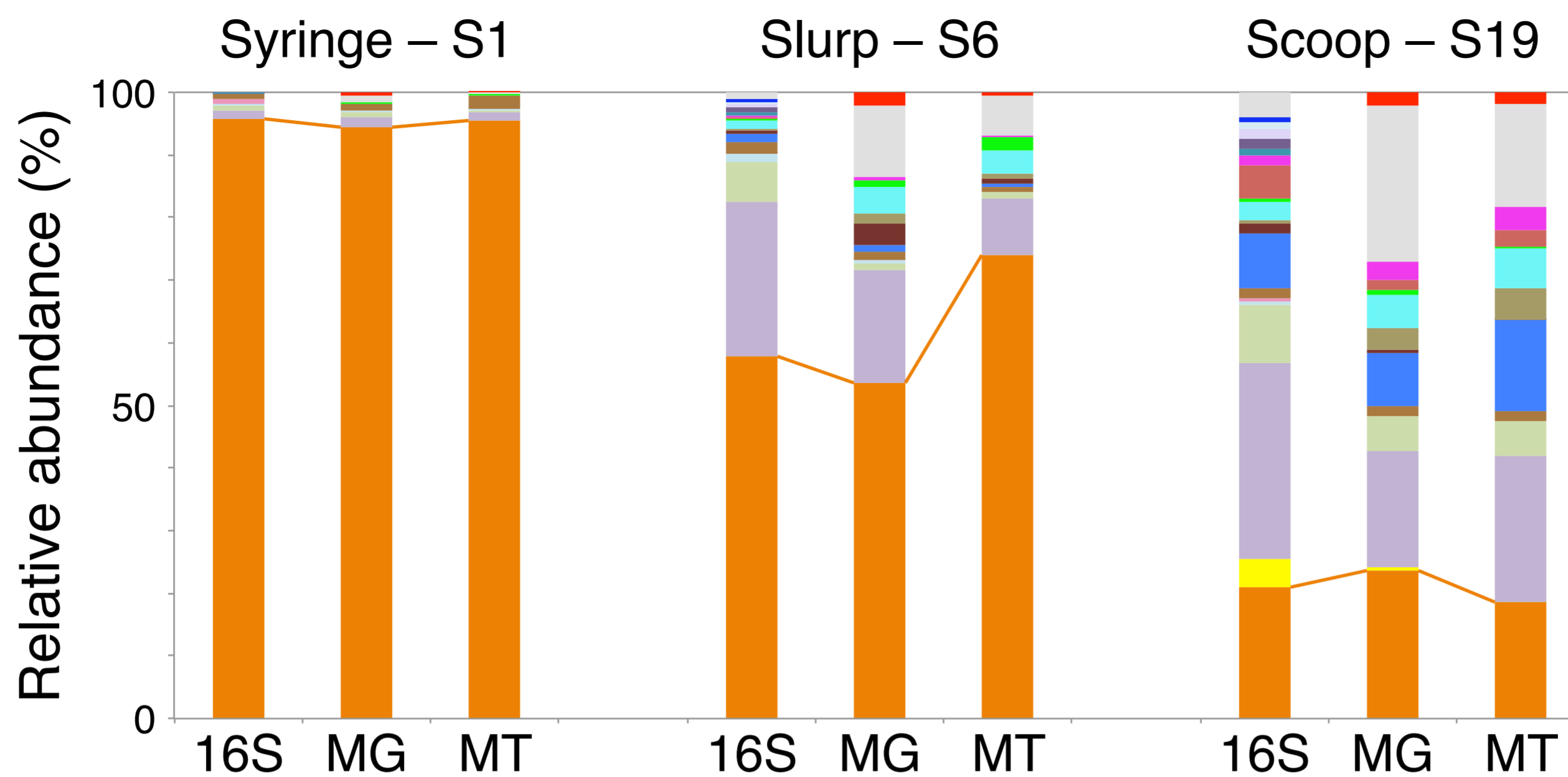

**B** MAR

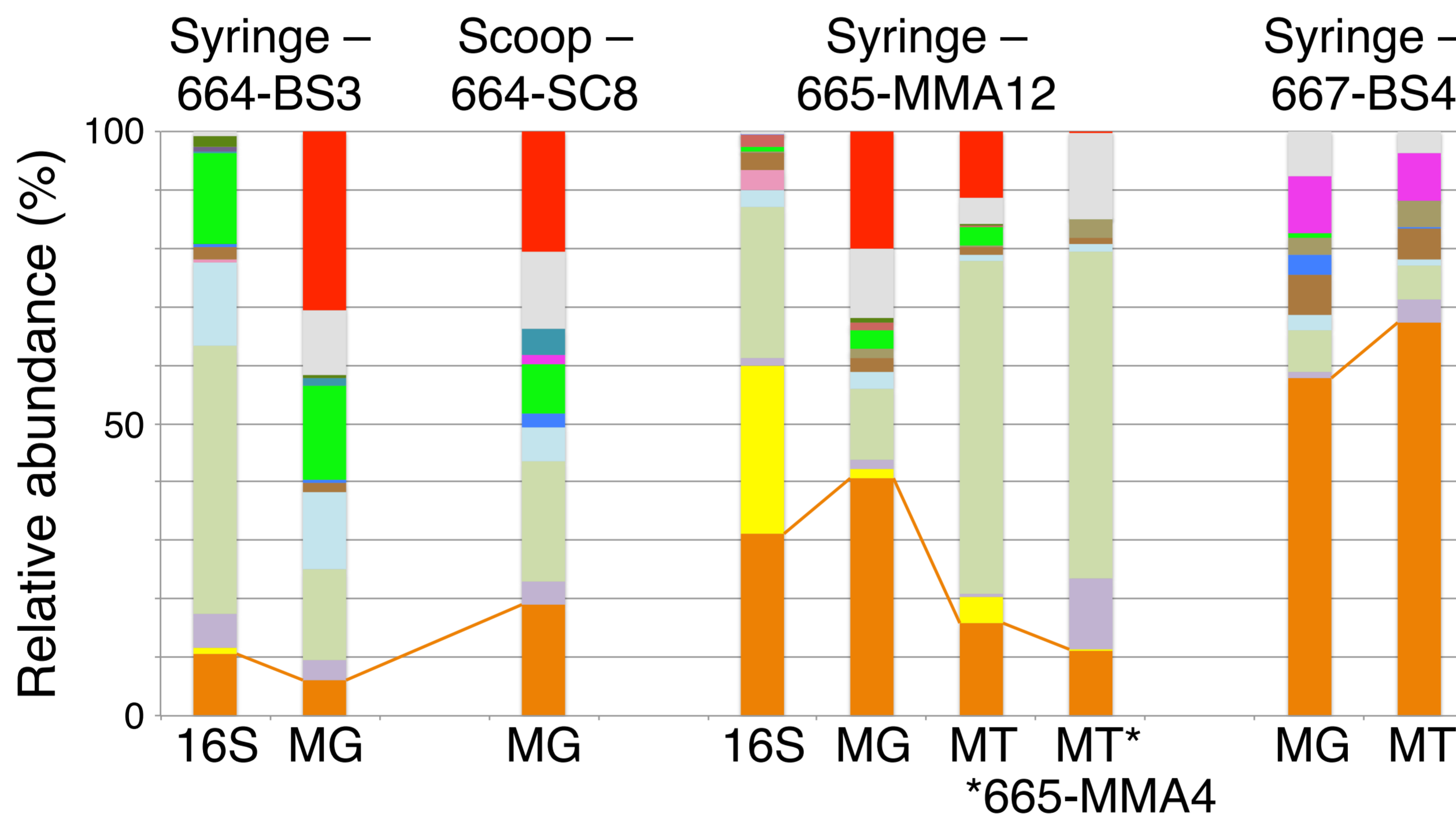

**C** Mariana

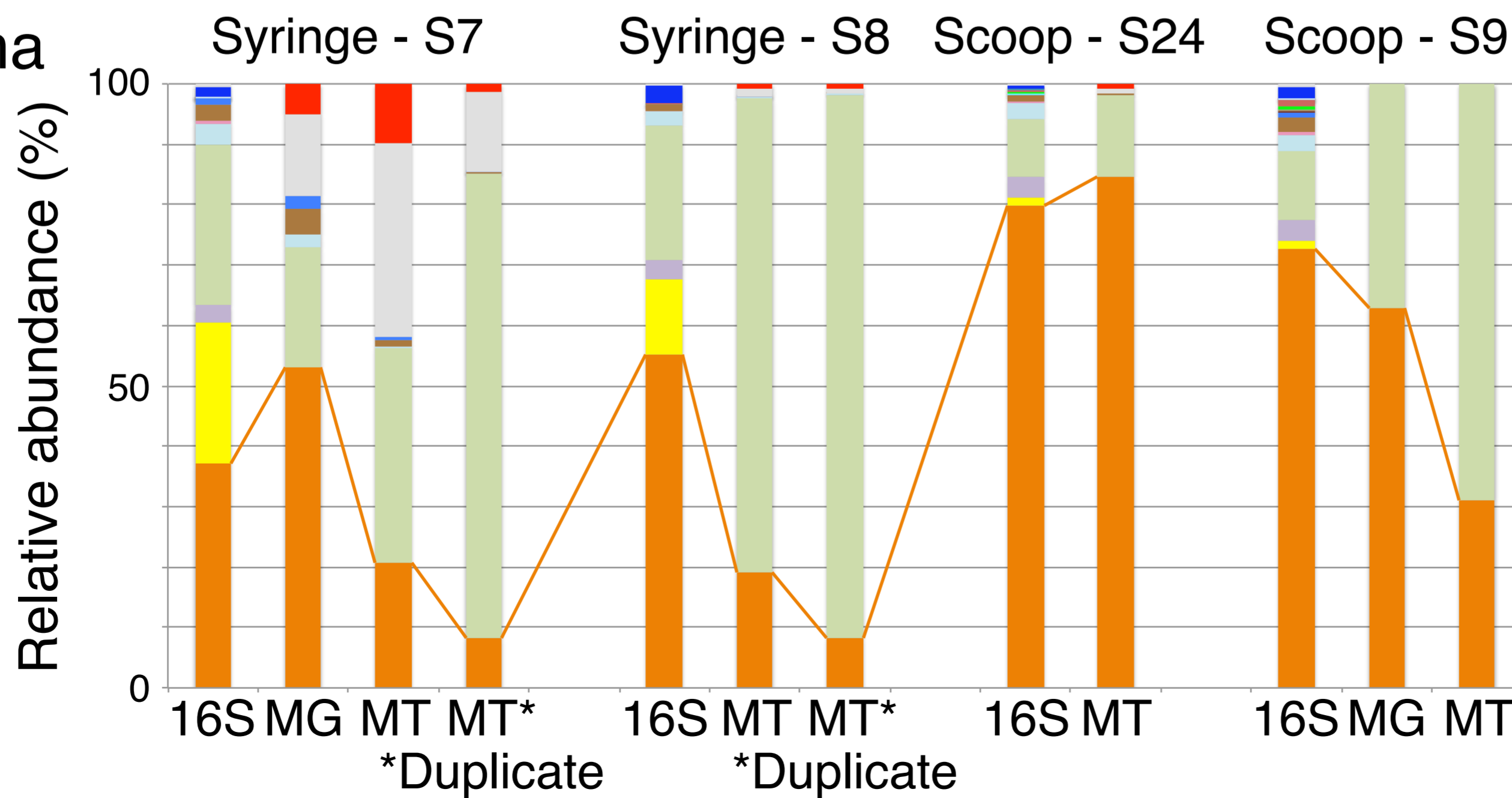

Supplement: FIG S4 [file mSystems.00553-19-sf004.pdf]

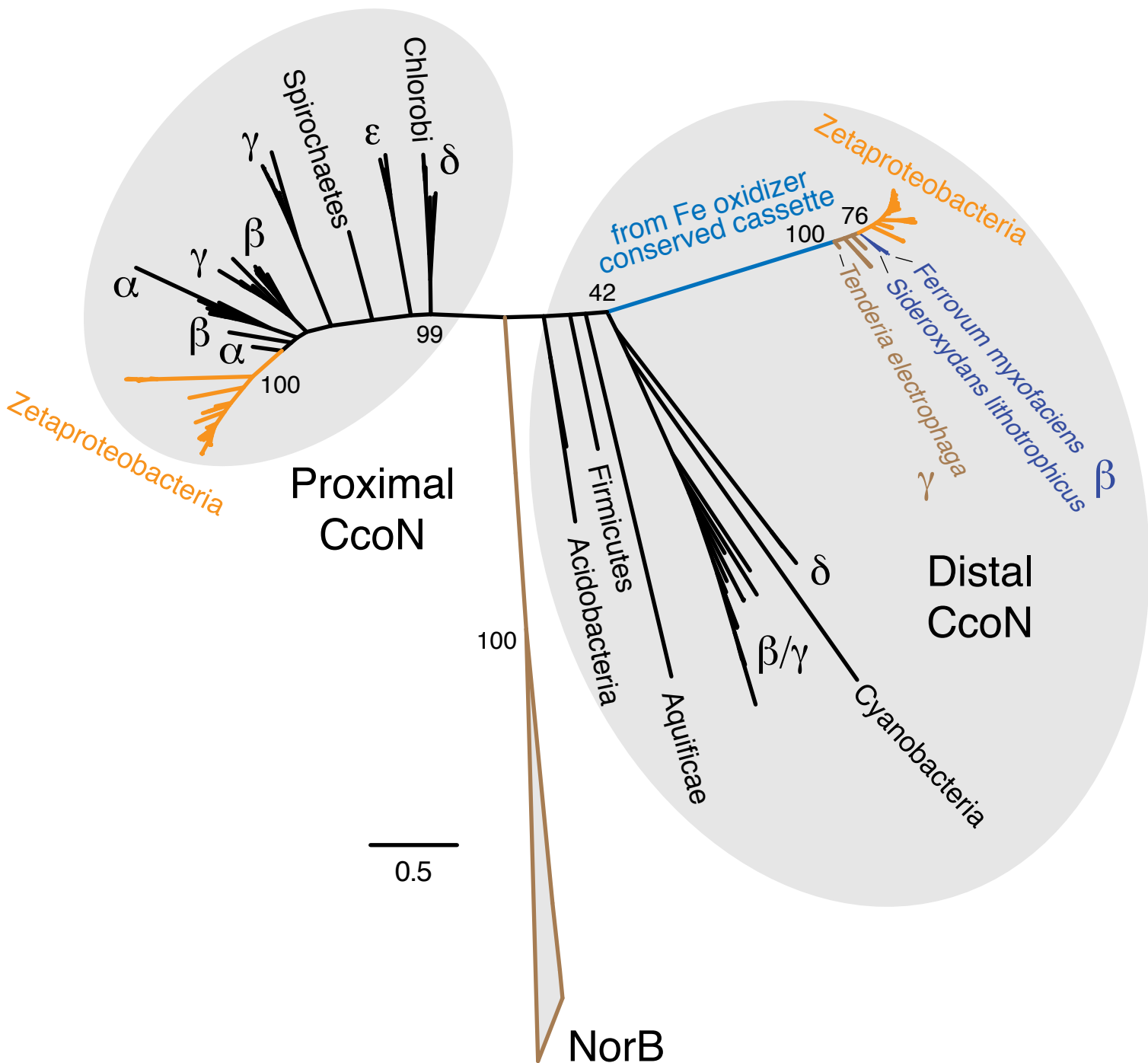

Supplement: FIG S5 [file mSystems.00553-19-sf005.pdf]

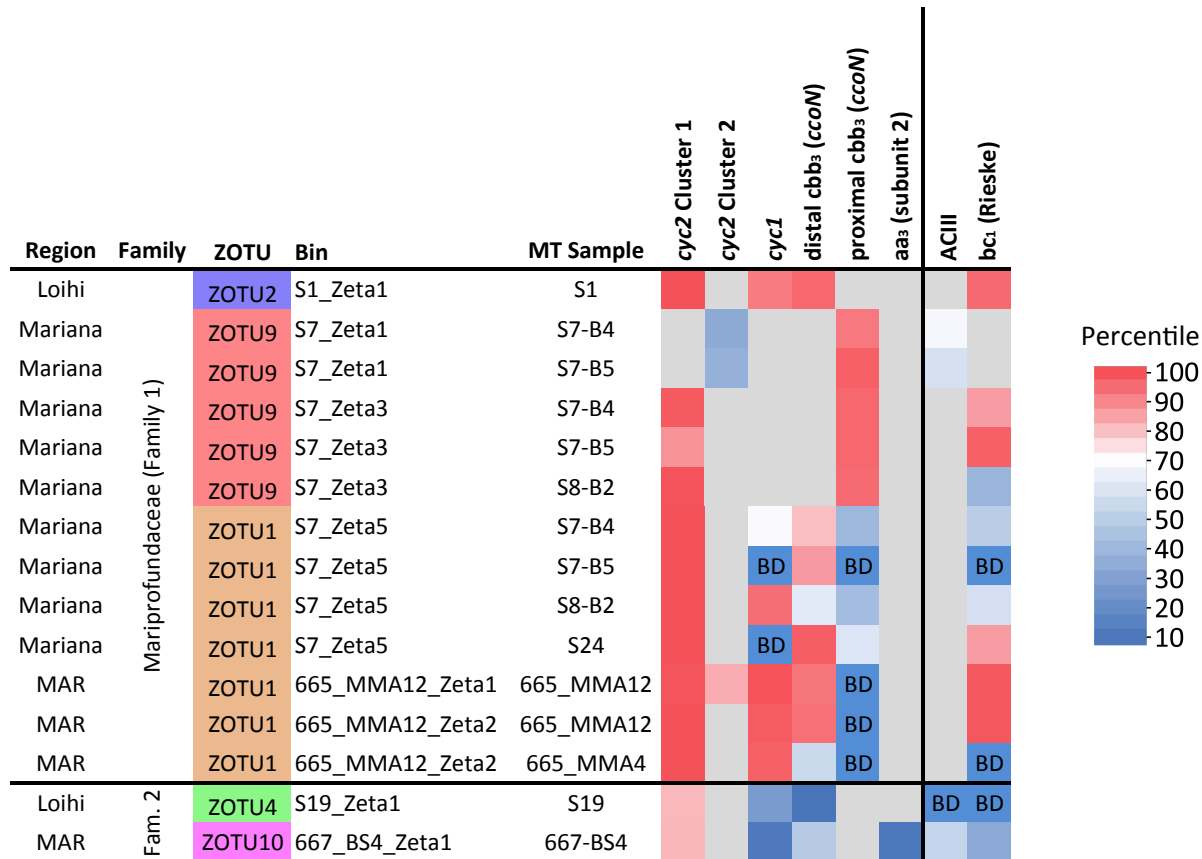

Supplement: FIG S6 [file mSystems.00553-19-sf006.pdf]
