# Supplementary material for: Validating the Cyc2 Neutrophilic Iron Oxidation Pathway Using Meta-omics of Zetaproteobacteria Iron Mats at Marine Hydrothermal Vents
Source: mSystems. 2020 Feb 18;5(1):e00553-19. doi: 10.1128/mSystems.00553-19 (PMC7029218; doi:10.1128/mSystems.00553-19)
Supplement: FIG S7 [file mSystems.00553-19-sf007.pdf]

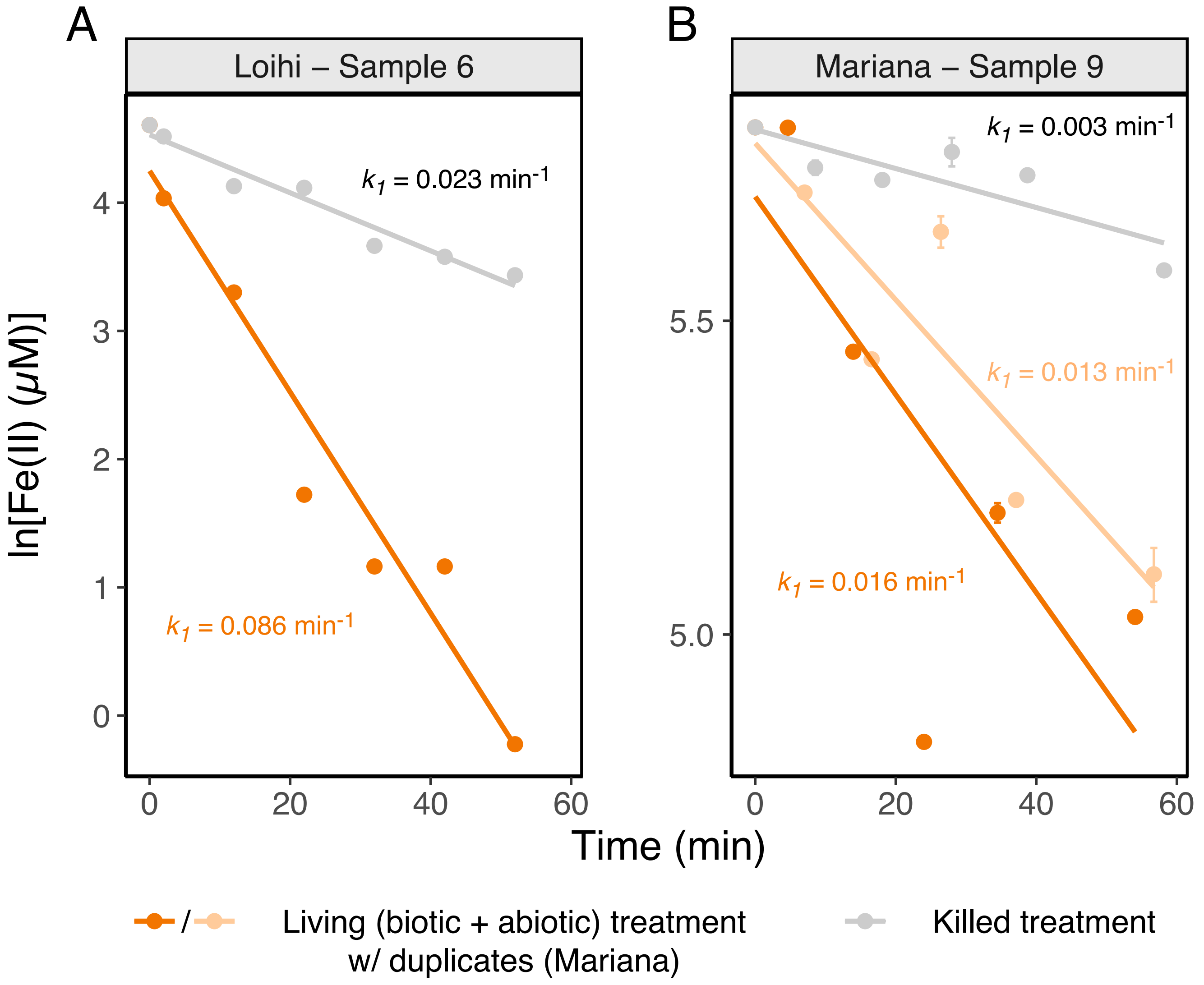
A
Loihi – Sample 6
k_1 = 0.023 min^{-1}
k_1 = 0.086 min^{-1}
B
Mariana – Sample 9
k_1 = 0.003 min^{-1}
k_1 = 0.013 min^{-1}
k_1 = 0.016 min^{-1}
ln[Fe(II) (μM)]
Time (min)
0
20
40
60
0
1
2
3
4
5.0
5.5
Living (biotic + abiotic) treatment w/ duplicates (Mariana)
Killed treatment
